# Supplementary material for: Breathing porous liquids based on responsive metal-organic framework particles
Source: Nat Commun. 2023 Jul 14;14:4200. doi: 10.1038/s41467-023-39887-3 (PMC10349080; doi:10.1038/s41467-023-39887-3)
Supplement: Supplementary file 1 — Supplementary Information File [file 41467_2023_39887_MOESM1_ESM.pdf]

Supplementary Information

Breathing porous liquids based on responsive metal-organic framework particles

Athanasios Koutsianos[1], Roman Pallach[1], Louis Frentzel-Beyme[1], Chinmoy Das[1], Michael Paulus[2], Christian Sternemann[2], Sebastian Henke[1*]

[1] Anorganische Chemie, Fakultät für Chemie und Chemische Biologie, Technische Universität Dortmund, Otto-Hahn-Straße 6, 44227 Dortmund, Germany. Email: sebastian.henke@tu-dortmund.de

[2] Fakultät Physik/DELTA, Technische Universität Dortmund, Maria-Goeppert-Mayer Str. 2, 44221 Dortmund, Germany.

Contents

Supplementary Methods-Gas sorption measurements....2
Supplementary Methods-In situ XRD....3
Supplementary Figures-Powder X-ray diffraction....4
Supplementary Figures-Scanning electron microscopy....8
Supplementary Figures-Transmission electron microscopy....10
Supplementary Figures-Viscosity measurements....11
Supplementary Figures-DLS measurements....12
Supplementary Figures-^{1}H NMR spectroscopy....13
Supplementary Figures-Stability of PLs....15
Supplementary Figures-Thermogravimetric analysis....17
Supplementary Notes-Gas sorption measurements....21
Supplementary Figures-Gas sorption measurements....25
Supplementary Figures-In situ XRD....33
Supplementary Tables....39
Supplementary References....41

Supplementary Methods-Gas sorption measurements

In order to perform gas uptake measurements of the PLs under continuous stirring, the porosimeter, originally manufactured for the analysis of porous solids, was modified. A stirring plate was placed on the lift drive platform in place of the Dewar, and a jacketed glass flask was mounted on it and connected to a circulator to control the temperature. A float level sensor was used to control the height of the glass flask. 0.5 mL sample was injected into a 9 mm sample cell with large bulb and a PTFE stirring bar was added. The liquids (PLs and pure Silicone 704) were kept stirring during all measurements (except for the one dedicated CO_2 sorption experiment of **PL7_10** without stirring).

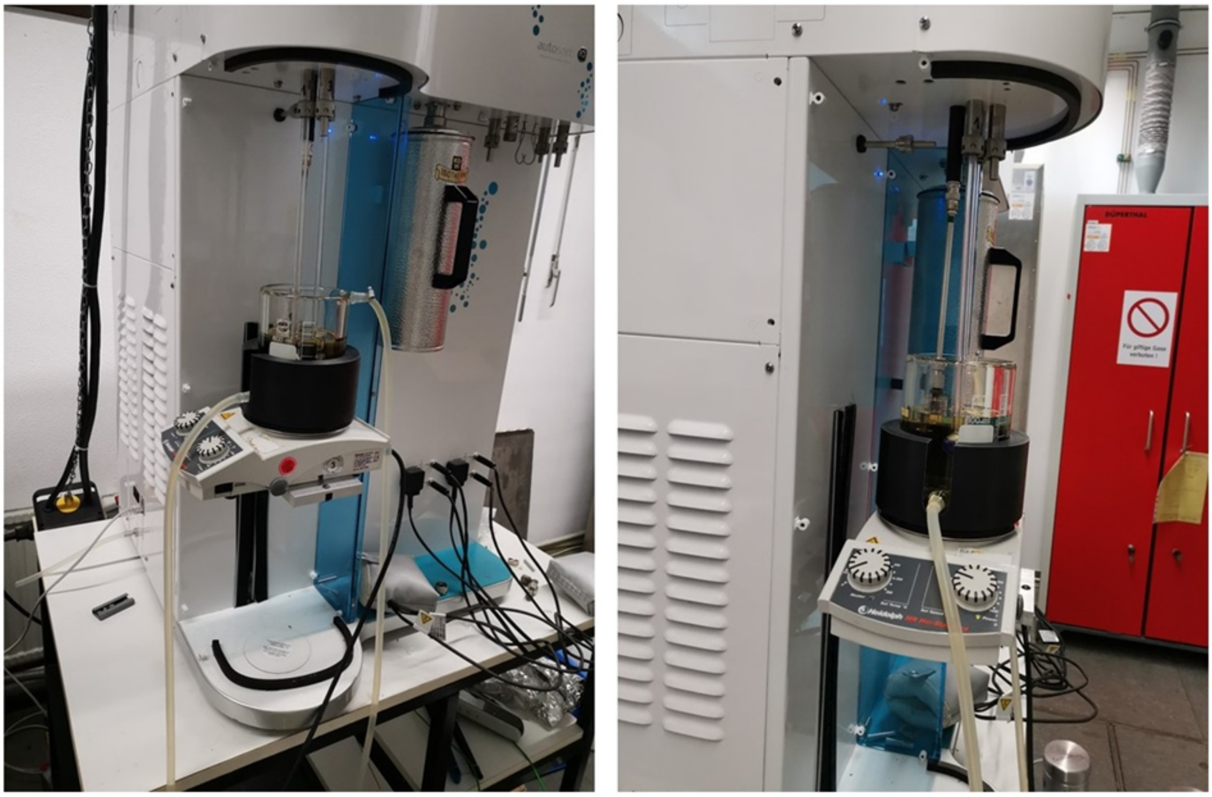

Supplementary Figure 1: Quantachrome Autosorb iQ MP instrument and the setup used for gas uptake measurements of PLs.

In the measurement routine of the Quantachrome Autosorb iQ MP a sample is considered to have reached equilibrium when the pressure change (Δp) is less than 0.81 mbar for a selected time interval (Δt) termed as *equilibration time* in the instrument software. Thus, the equilibration criterion is met when the magnitude of the pressure gradient is less than $\Delta p/\Delta t$ = 0.81 mbar/Δt). The equilibrium settings were the following: Δt = 12 min, pressure tolerance value of 1, i.e. 0.006-0.002 mbar for pressure values of 0.012-12.266 mbar, 0.074-0.024 mbar for pressure values from 12.266-306.641 mbar and 7.359-2.453 mbar for pressure values from 306.641-1226.562 mbar.

Supplementary Methods-In situ XRD

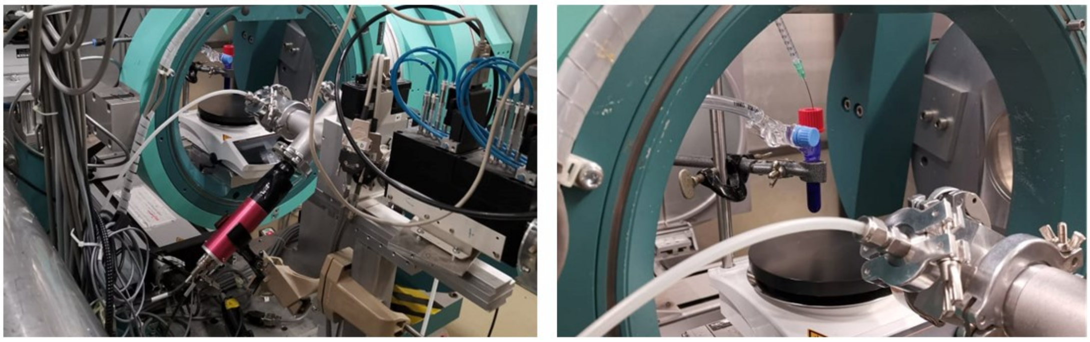

Supplementary Figure 2: Experimental setup used at DELTA facilities for in situ XRD experiments during gas sorption by the PLs.

Supplementary Figures-Powder X-ray diffraction

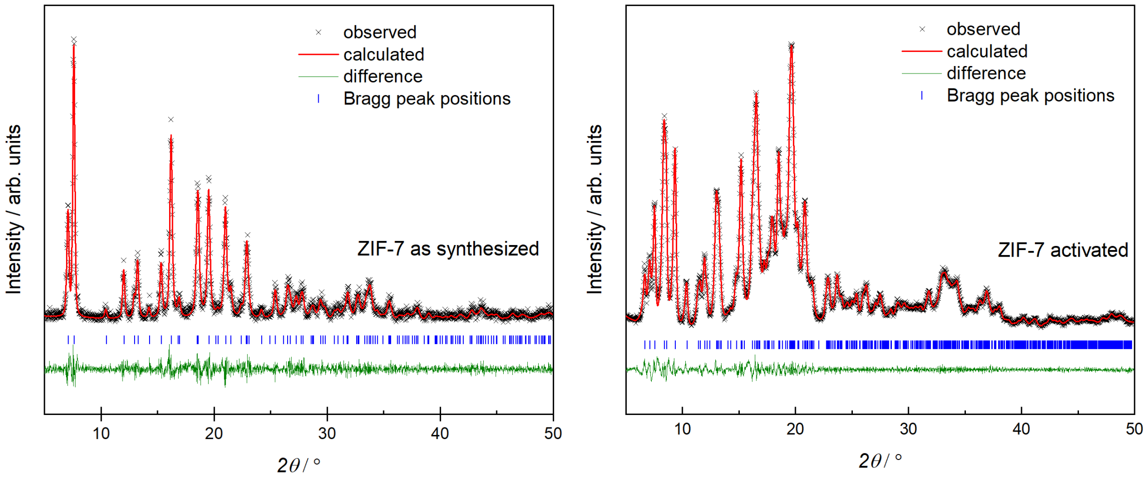

Supplementary Figure 3: PXRD patterns (λ = 1.5418 Å) with profile fits (Pawley method) of as synthesized and activated ZIF-7 nanocrystals. Crystallographic parameters of reference crystal structures from the literature were used as starting parameters for the profile fits (CCDC code VELVIS[1] for the as synthesized ***lp*** phase and CCDC code RIPNOV01[2] for the activated ***np*** phase).

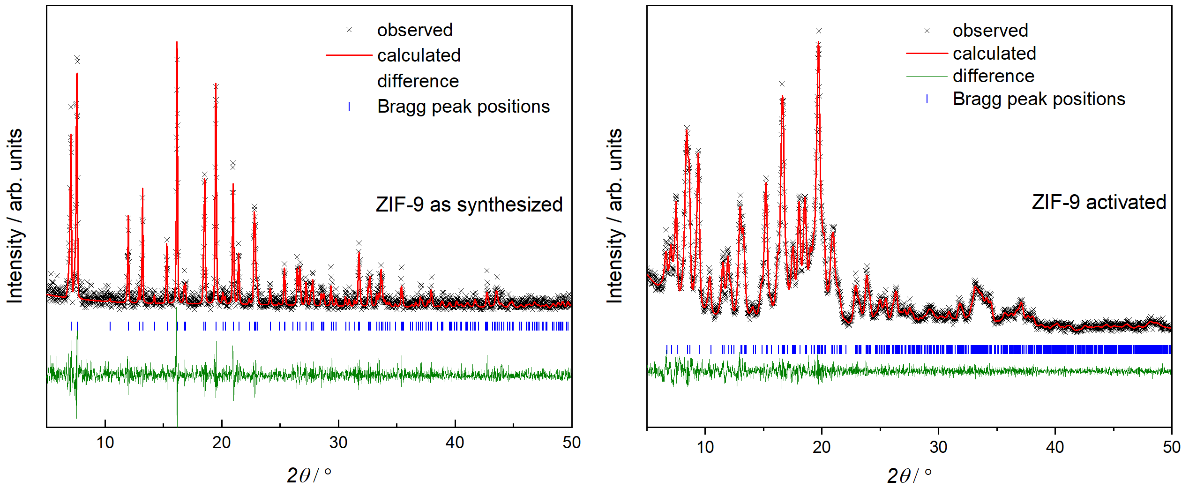

Supplementary Figure 4: PXRD patterns (λ = 1.5418 Å) with profile fits (Pawley method) of as synthesized and activated ZIF-9 nanocrystals. Crystallographic parameters of reference crystal structures from the literature were used as starting parameters for the profile fits (CCDC code VEJZEQ[1] for the as synthesized ***lp*** phase and CCDC code RIPNOV01[2] for the activated ***np*** phase).

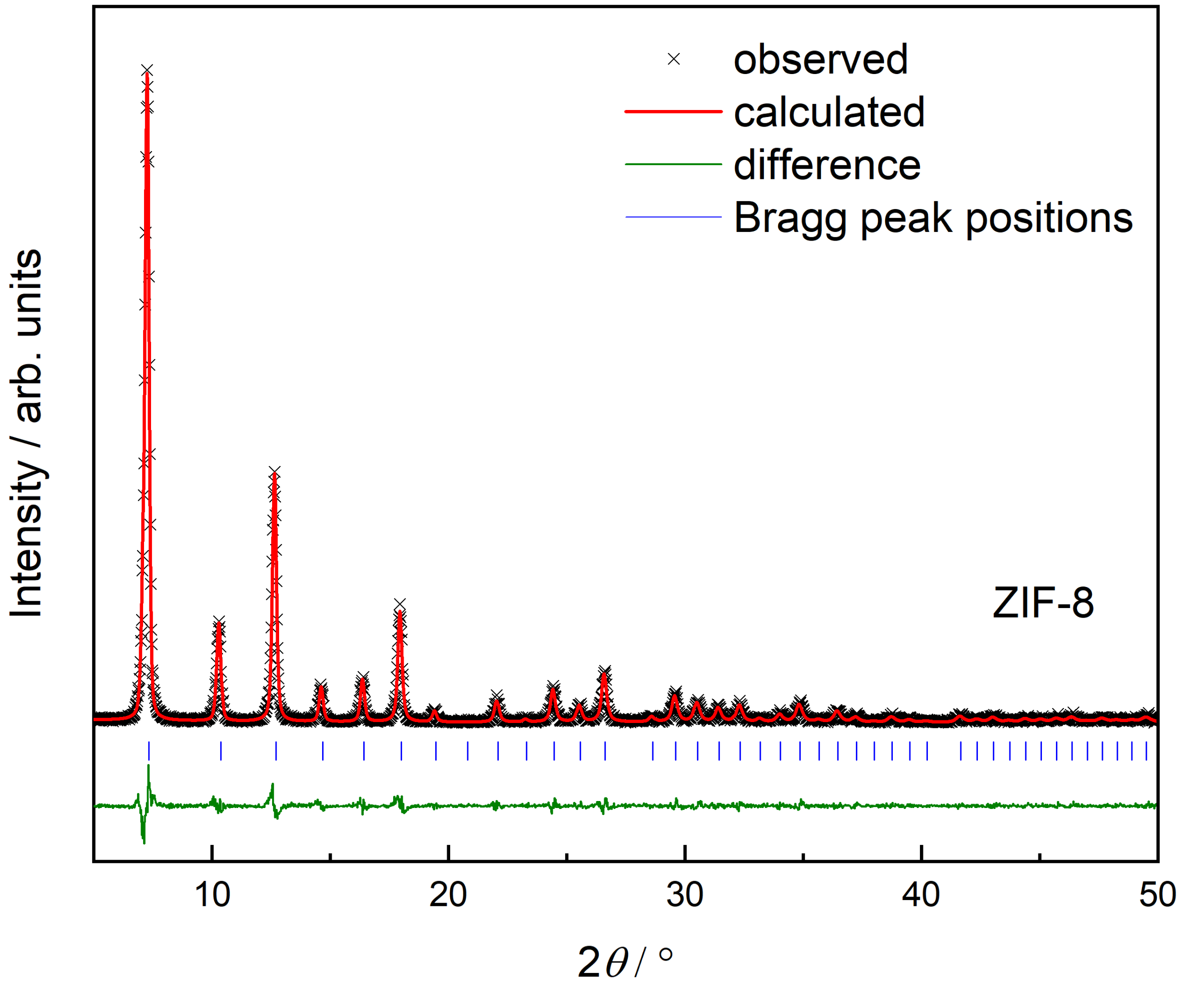

Supplementary Figure 5: PXRD pattern (λ = 1.5418 Å) with profile fit (Pawley method) of ZIF-8 nanocrystals. Crystallographic parameters of the reference crystal structure from the literature were used as starting parameters for the profile fit (CCDC code VELVOY[1]).

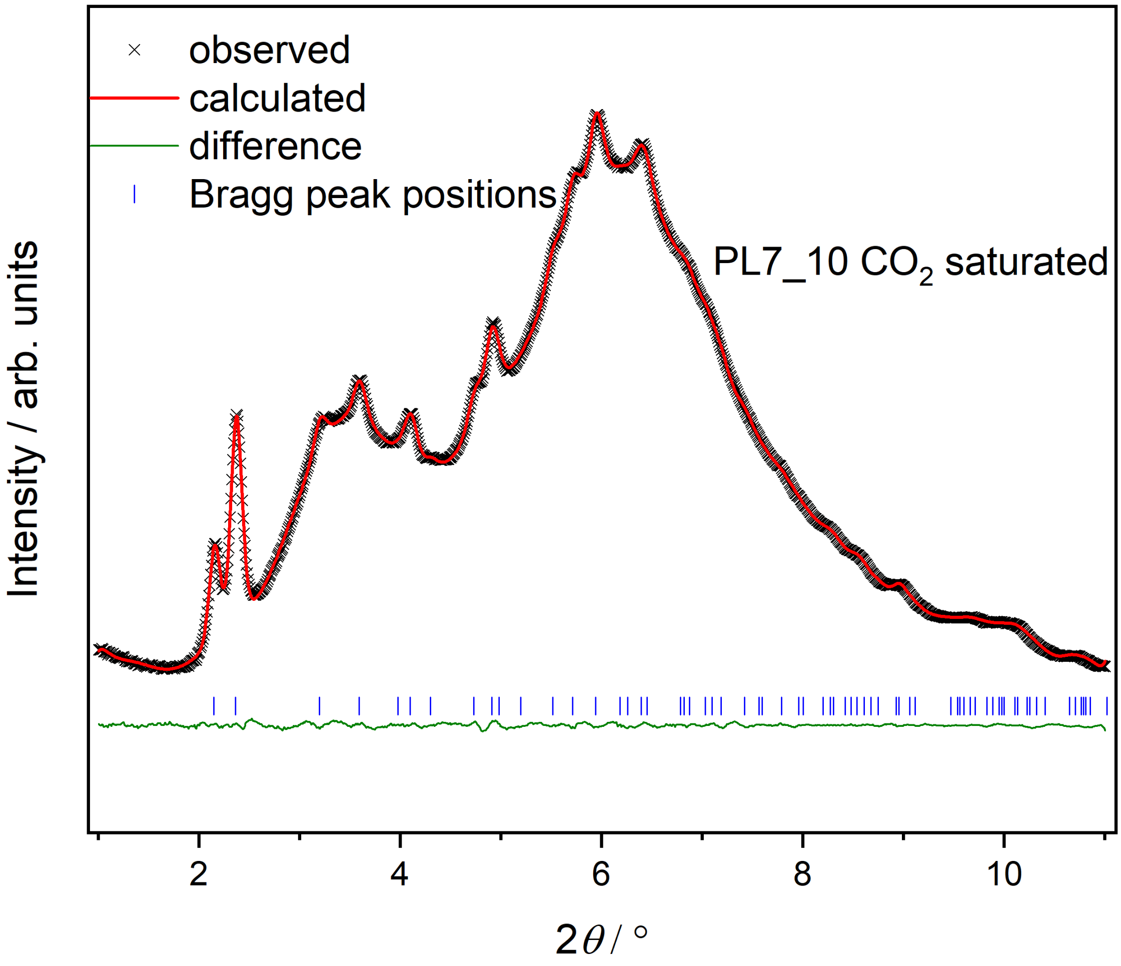

Supplementary Figure 6: PXRD pattern (λ = 0.45904 Å) with profile fit (Pawley method) of CO_2 saturated **PL7_10**. Crystallographic parameters of reference ZIF-7 crystal structure from the literature were used as starting parameters for the profile fit (CCDC code VEJZEQ[1]).

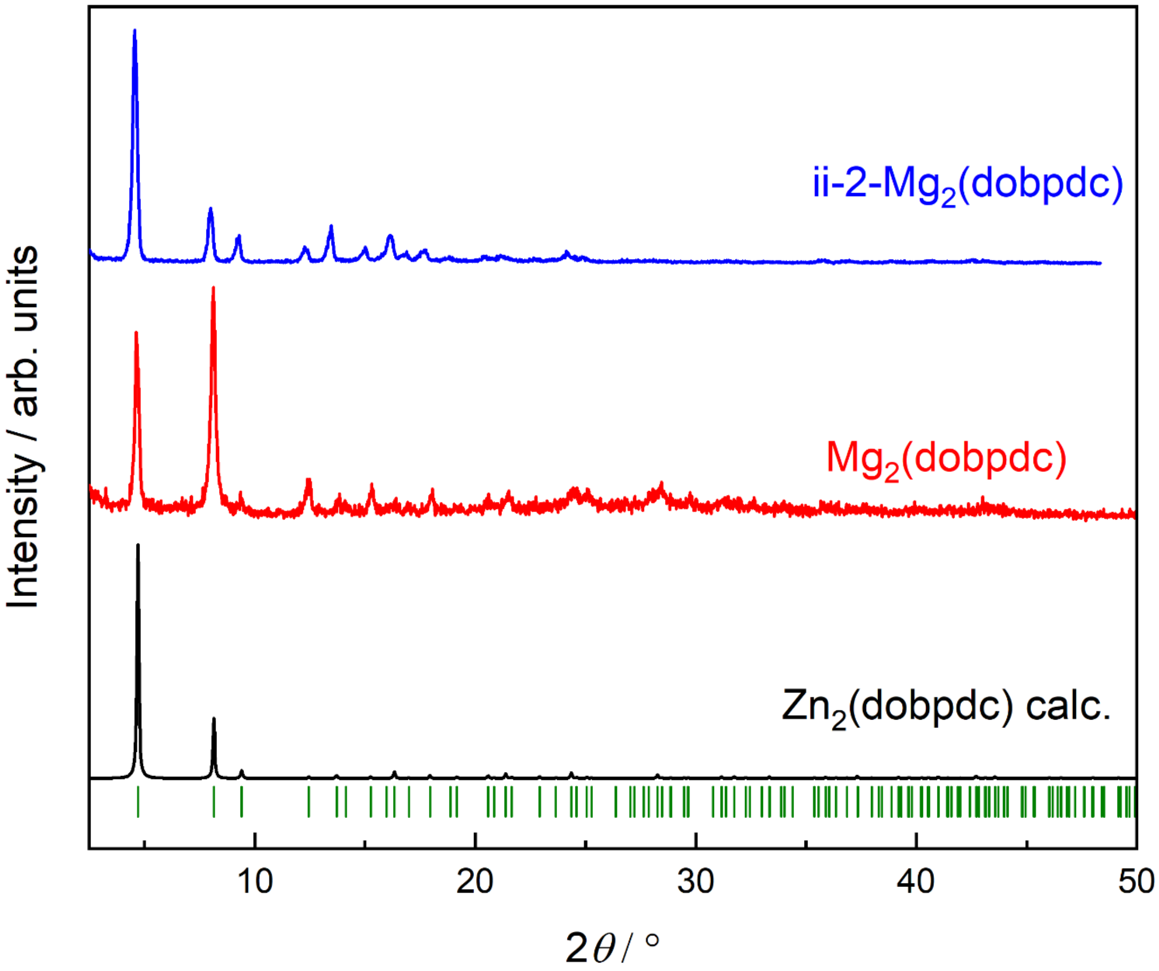

Supplementary Figure 7: PXRD patterns (λ = 1.5418 Å) of ii-2-Mg_2(dobpdc), Mg_2(dobpdc) and a calculated diffraction pattern of the isostructural Zn_2(dobpdc) (CCDC code XUXPUD)[3]. Green tick marks highlight the allowed Bragg peak positions of Zn_2(dobpdc).

Supplementary Figures-Scanning electron microscopy

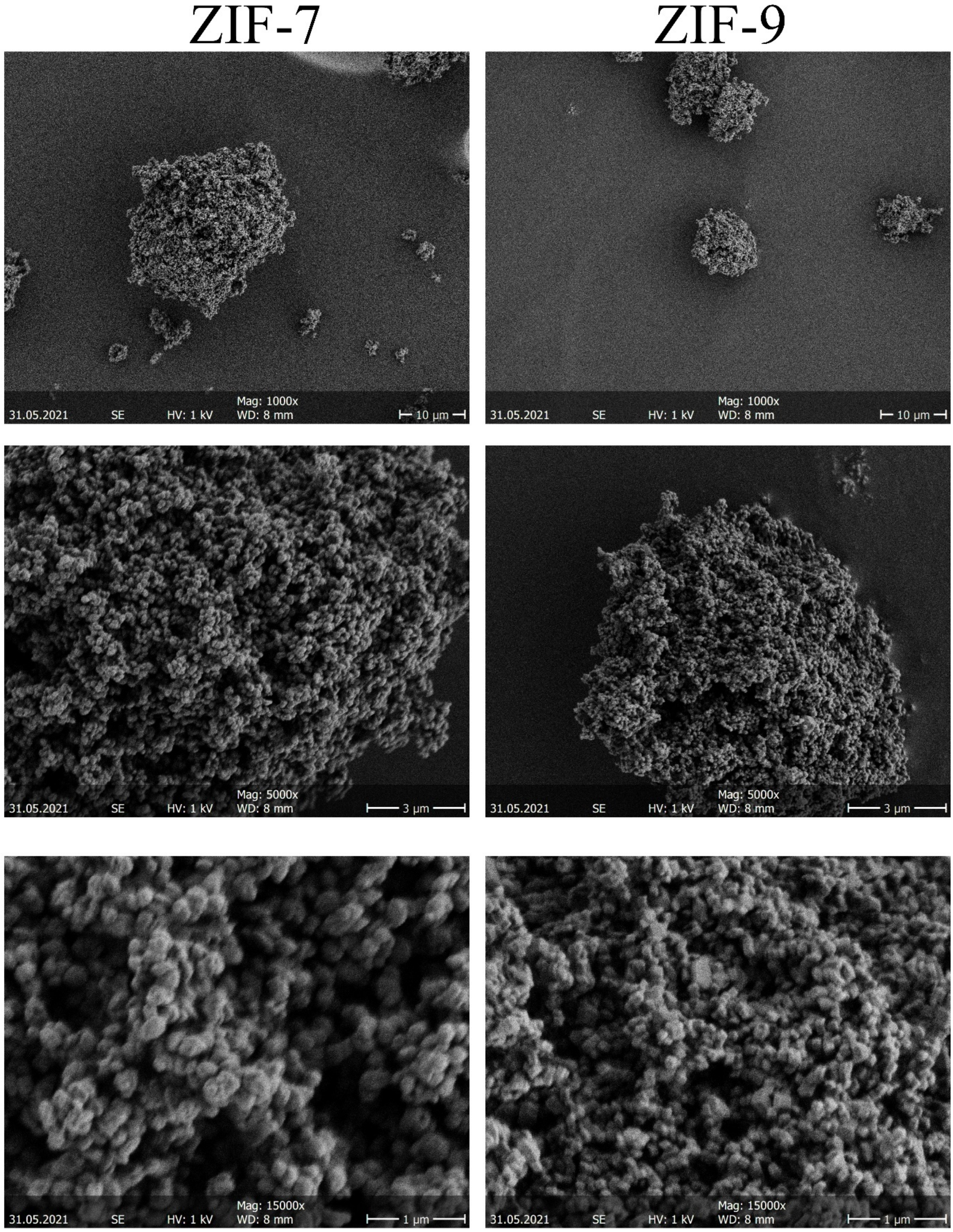

Supplementary Figure 8: SEM micrographs of ZIF-7 nanocrystals (top) and ZIF-9 nanocrystals (bottom) used for the development of PLs. The scale bar is depicted at the right bottom of the images.

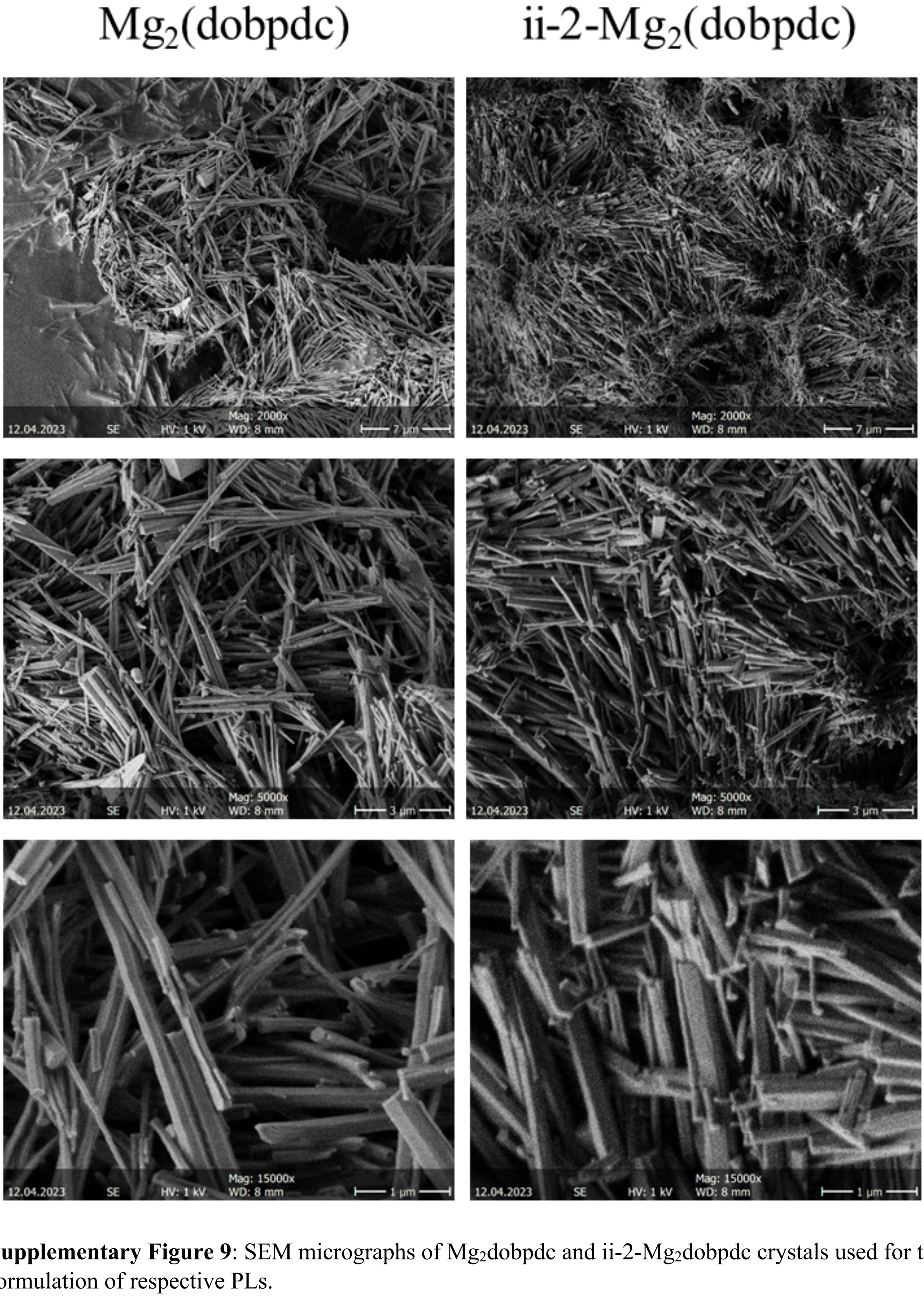

Supplementary Figure 9: SEM micrographs of Mg_2dobpdc and ii-2-Mg_2dobpdc crystals used for the formulation of respective PLs.

Supplementary Figures-Transmission electron microscopy

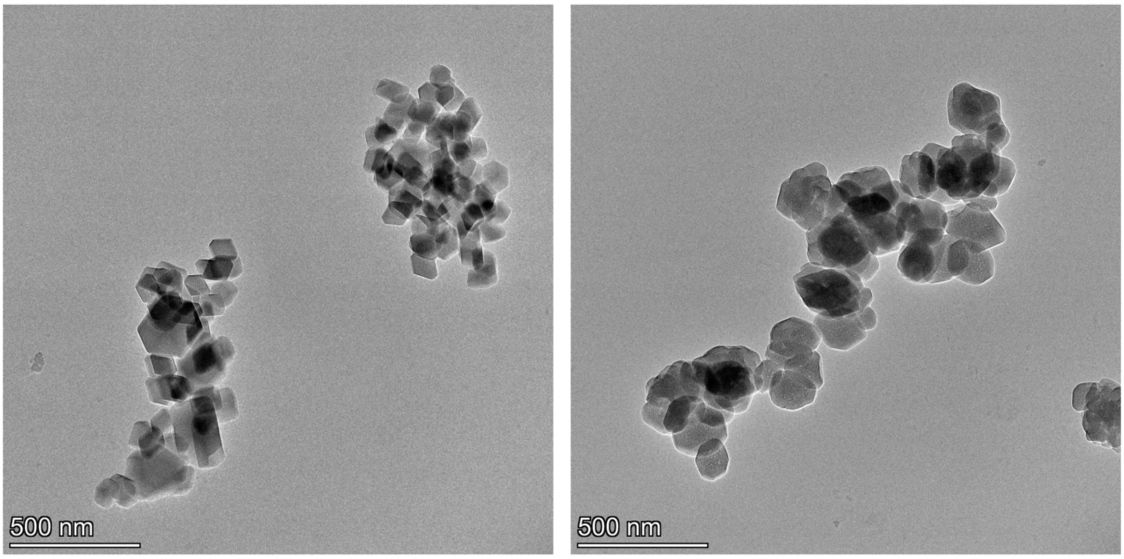

Supplementary Figure 10: TEM image of ZIF-7 (left) and ZIF-9 (right) nanocrystals used for the development of porous liquids.

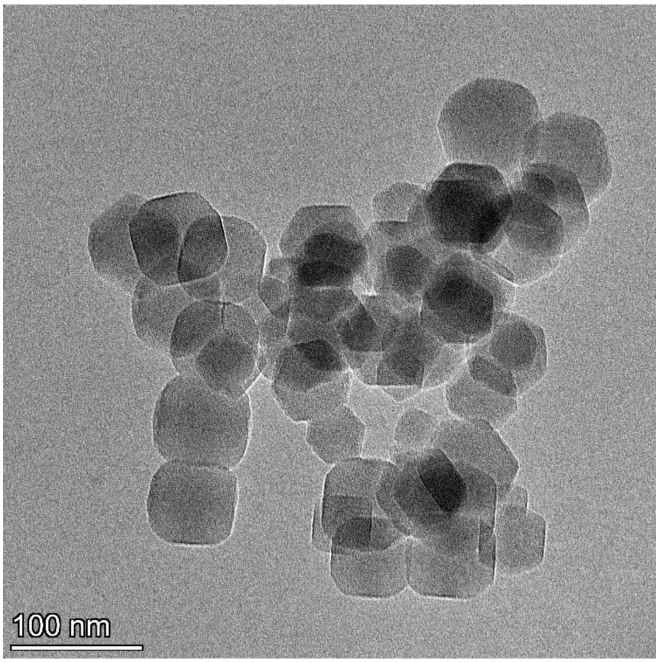

Supplementary Figure 11: TEM image of ZIF-8 nanocrystals used for the development of **PL8_5.**

Supplementary Figures-Viscosity measurements

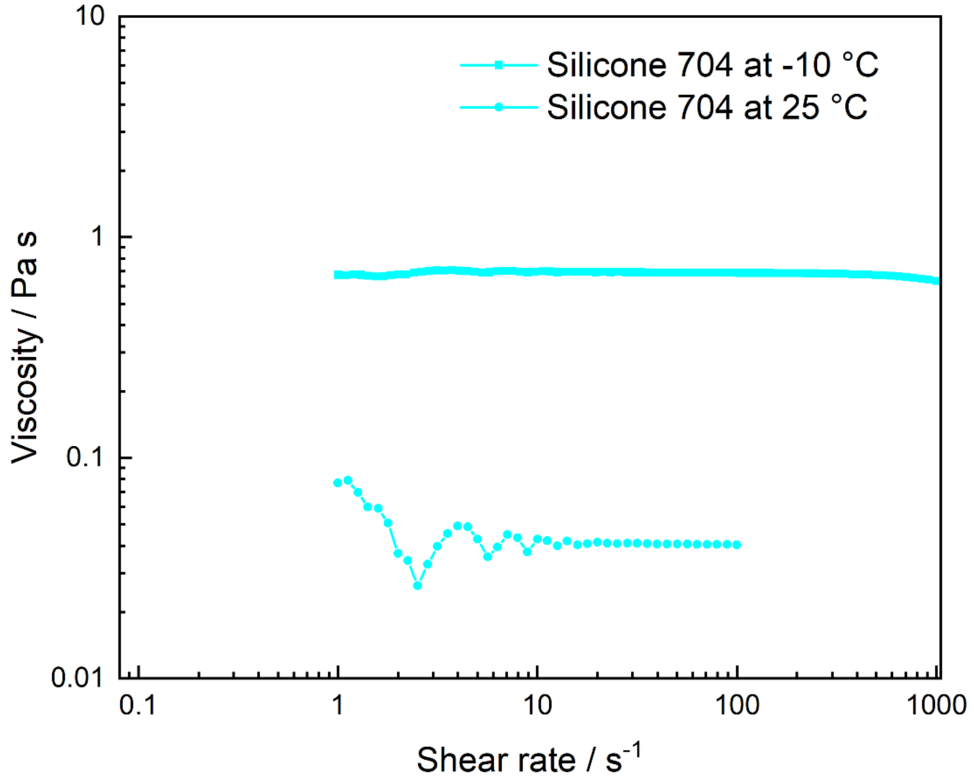

Supplementary Figure 12: Graph of the dynamic viscosity vs. shear rate for Silicone 704 at –10 °C. The respective measurement at 25 °C is also included for the sake of comparison. Clearly, the variations in the measured viscosity at 25 °C at shear rates below 10 s^{-1} must be ascribed to measurement errors and not to non-Newtonian behaviour. The measurement performed at –10 °C proves the Newtonian behaviour of Silicone 704.

Supplementary Figures-DLS measurements

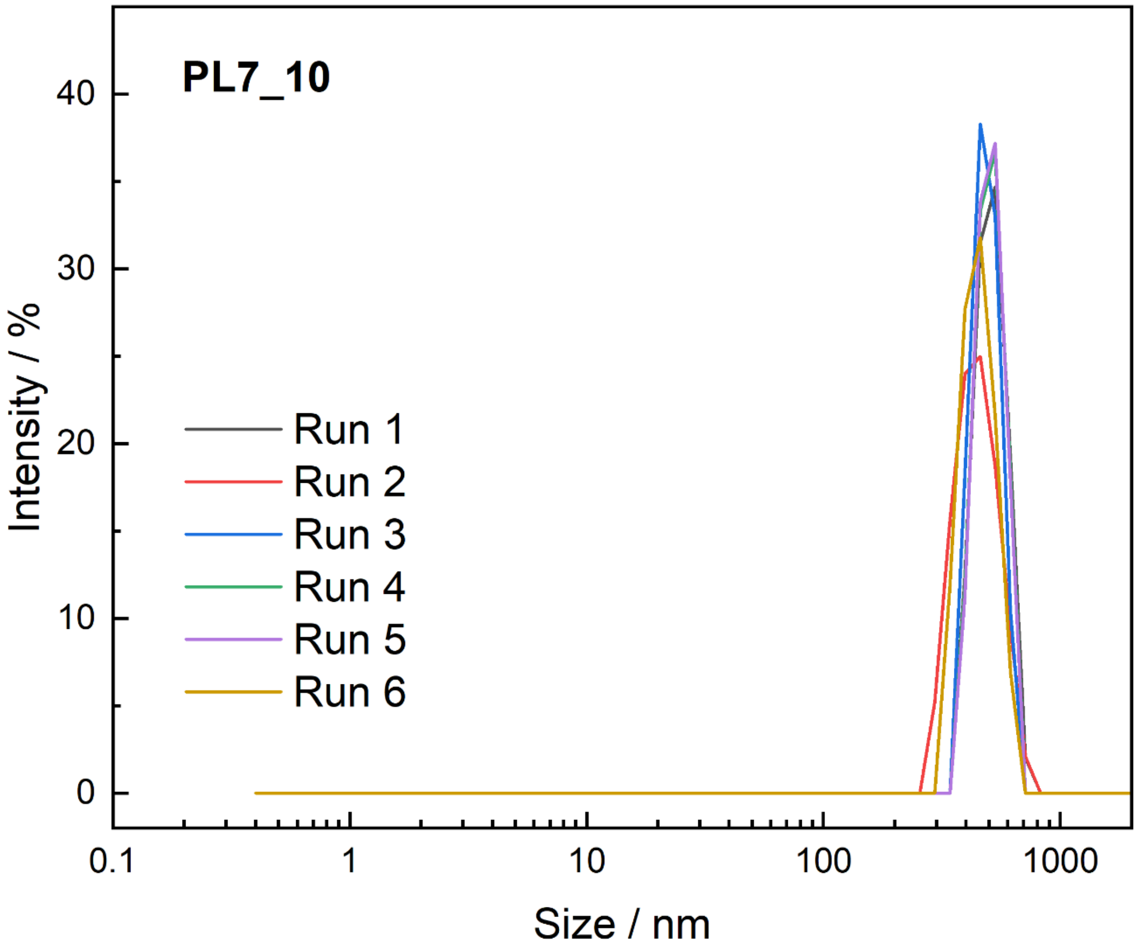

Supplementary Figure 13: DLS measurements of **PL7_10.** The sample was diluted 750-fold to obtain accurate DLS data. The calculated average particle size is 469 ± 74 nm with a polydispersity index of 0.058.

Supplementary Figures-^{1}H NMR spectroscopy

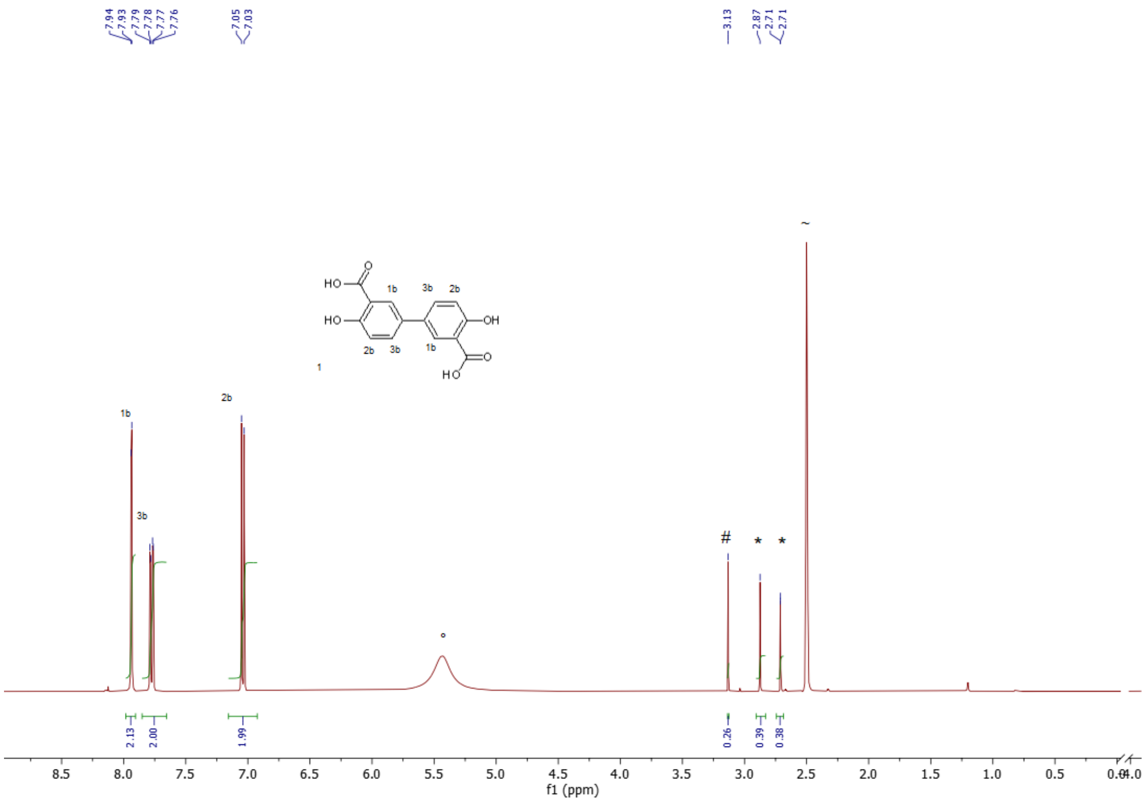

Supplementary Figure 14: ^{1}HNMR spectrum of a digested sample of as synthesized Mg_2(dobpdc). Residual proton signals from DMSO and D_2O are marked with ~ and °, respectively. Methanol and DMF guest molecules present in the framework are marked with # and *, respectively.

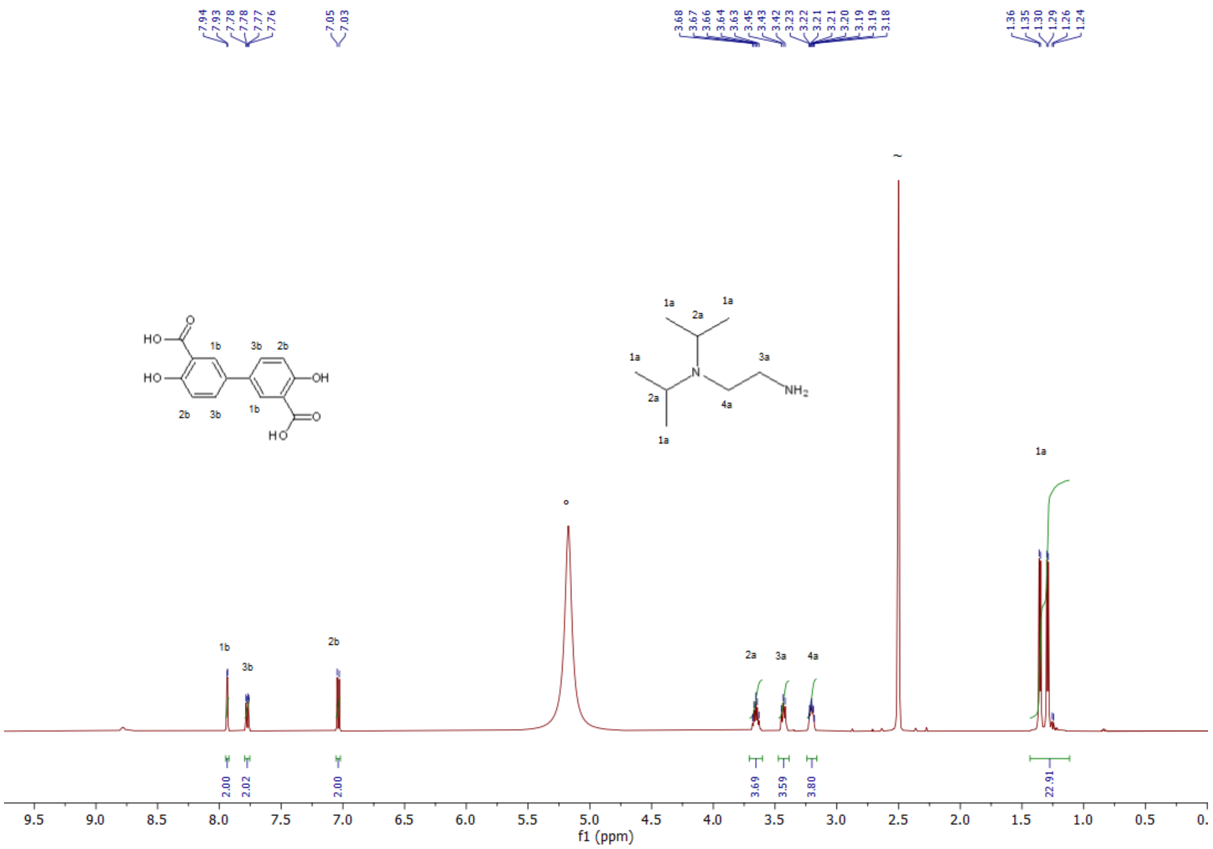

Supplementary Figure 15: ^{1}H NMR spectrum of a digested sample of ii-2-Mg_2(dobpdc). Residual proton signals from DMSO and D_2O are marked with ~ and °, respectively. Diamine loading is an average of the ratios calculated using each diamine signal and equals to 94 mol%, i.e. a chemical composition of (*N,N*-diisopropylethylenediamine)$_{1.88}$$Mg_2$(dobpdc).

Supplementary Figures-Stability of PLs

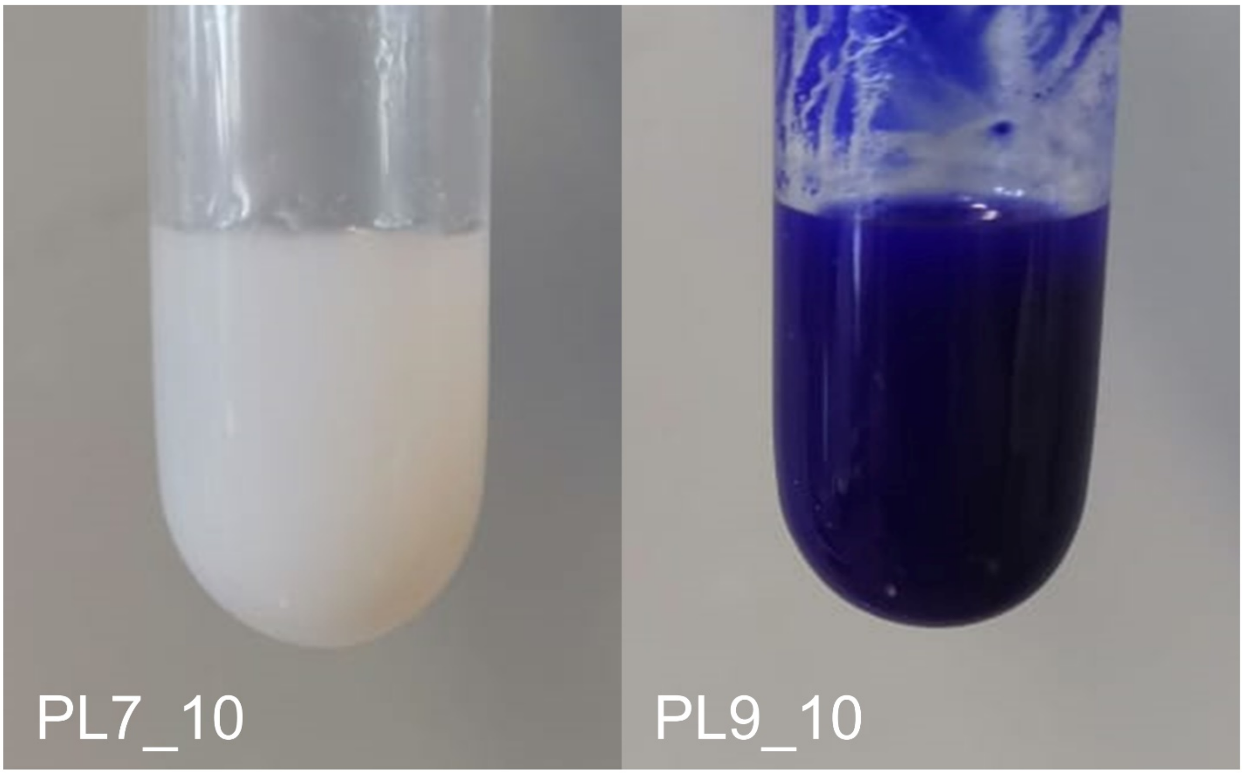

Supplementary Figure 16: Photographs of the formulated ZIF-based PLs after 3 months of storage without any agitation. The samples rested for 3 months in an isolated area in the lab. Sedimentation of the ZIF particle agglomerates is not observable.

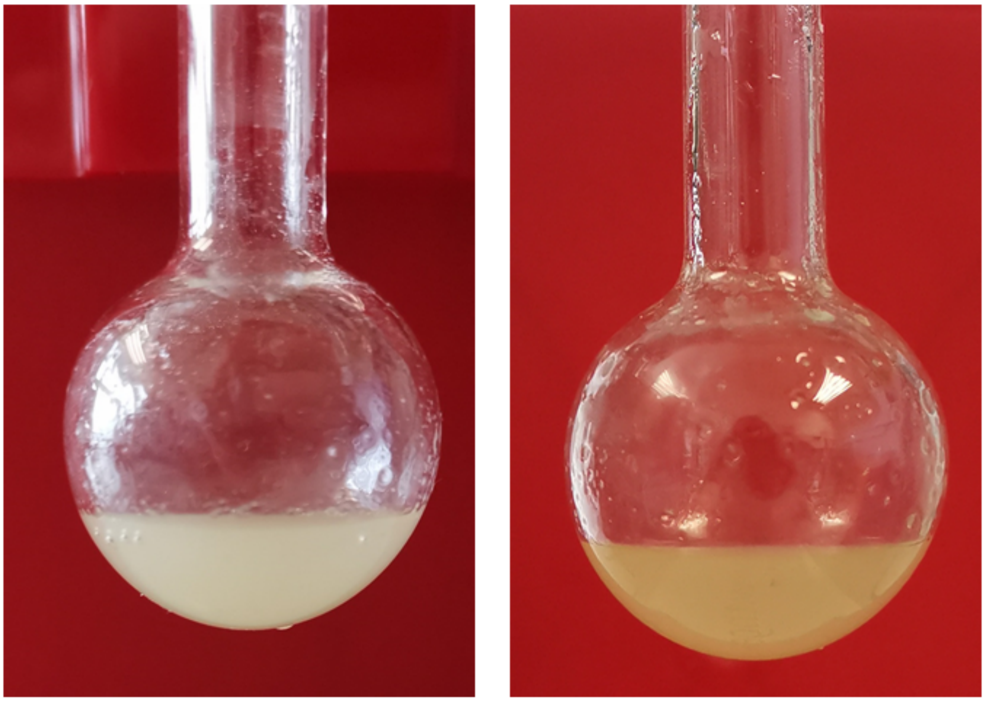

Supplementary Figure 17: Photographs of PL-ii-2-Mg_2(dobpdc) (5 wt% ii-2-Mg_2(dobpdc) in Silicone 704) directly after synthesis (left), after 12 days of storage (right). The sample was stored at ambient conditions without any agitation. Sedimentation of the MOF particles is not observable. The slight colour changes in PL are due to different lighting conditions in the laboratory.

Supplementary Figures-Thermogravimetric analysis

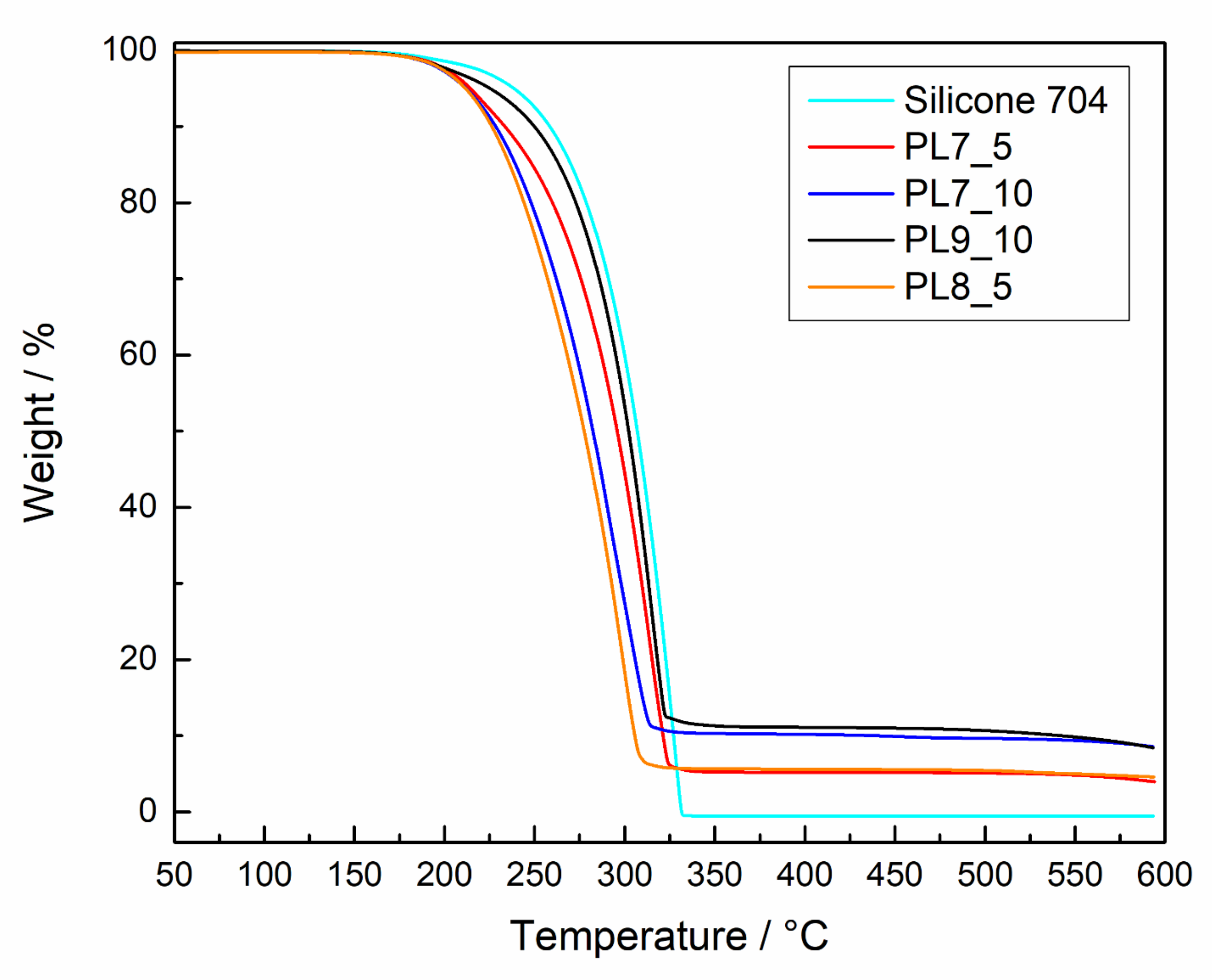

Supplementary Figure 18: TGA curves of Silicone 704, **PL7_5**, **PL7_10**, **PL9_10** and **PL8_5** under dynamic heating (10 °C min^{-1}) in N_2 atmosphere.

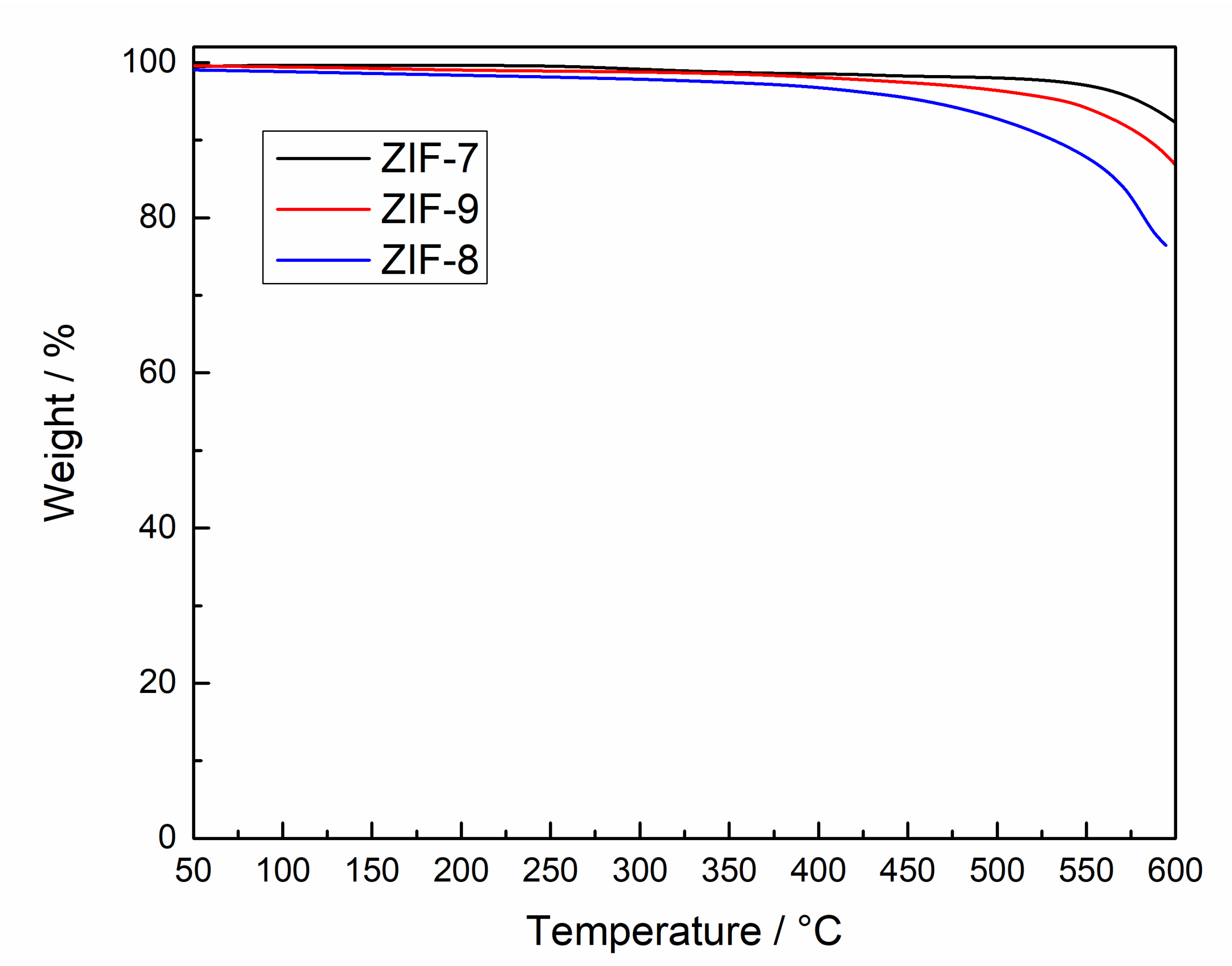

Supplementary Figure 19: TGA curves of ZIF-7, ZIF-9 and ZIF-8 nanocrystals under dynamic heating (10 °C min^{-1}) in N_2 atmosphere.

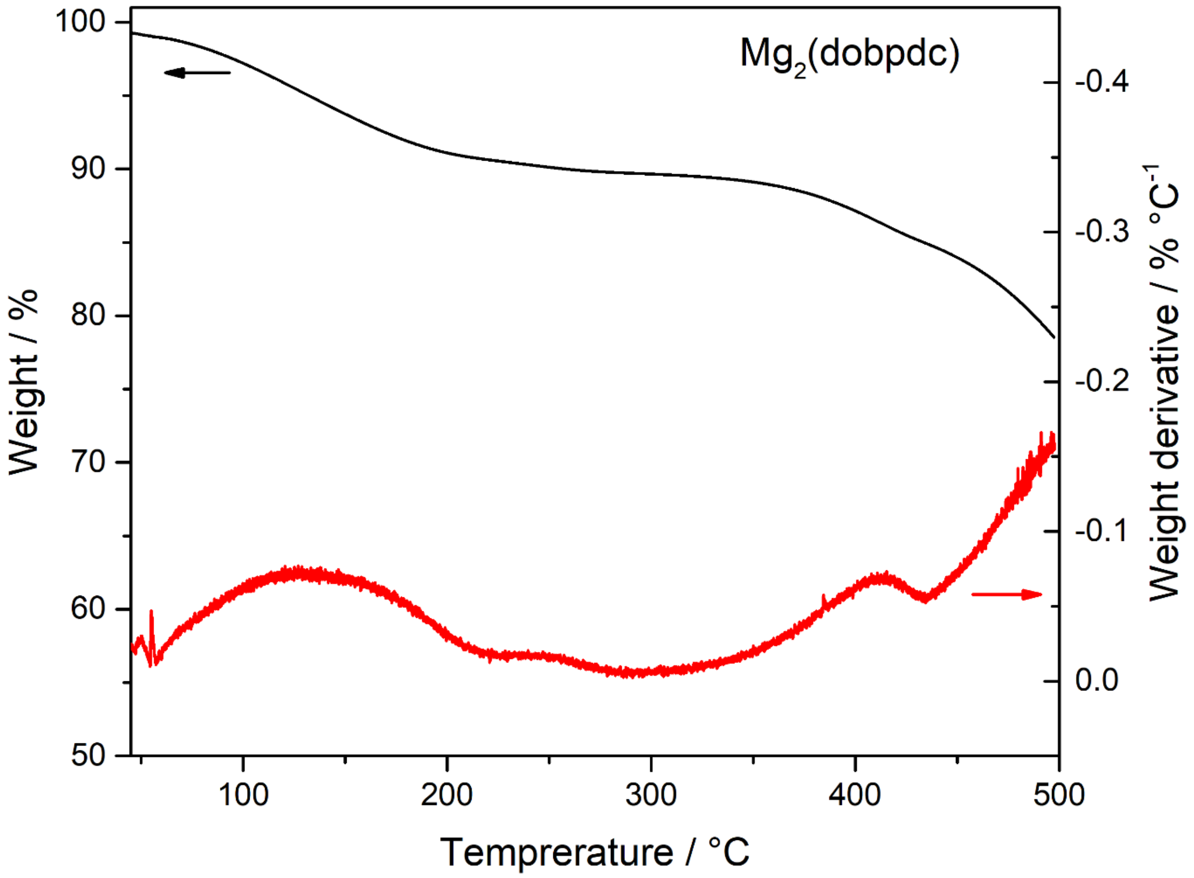

Supplementary Figure 20: TGA curve of Mg_2(dobpdc) under dynamic heating (3 °C min^{-1}) in N_2 atmosphere.

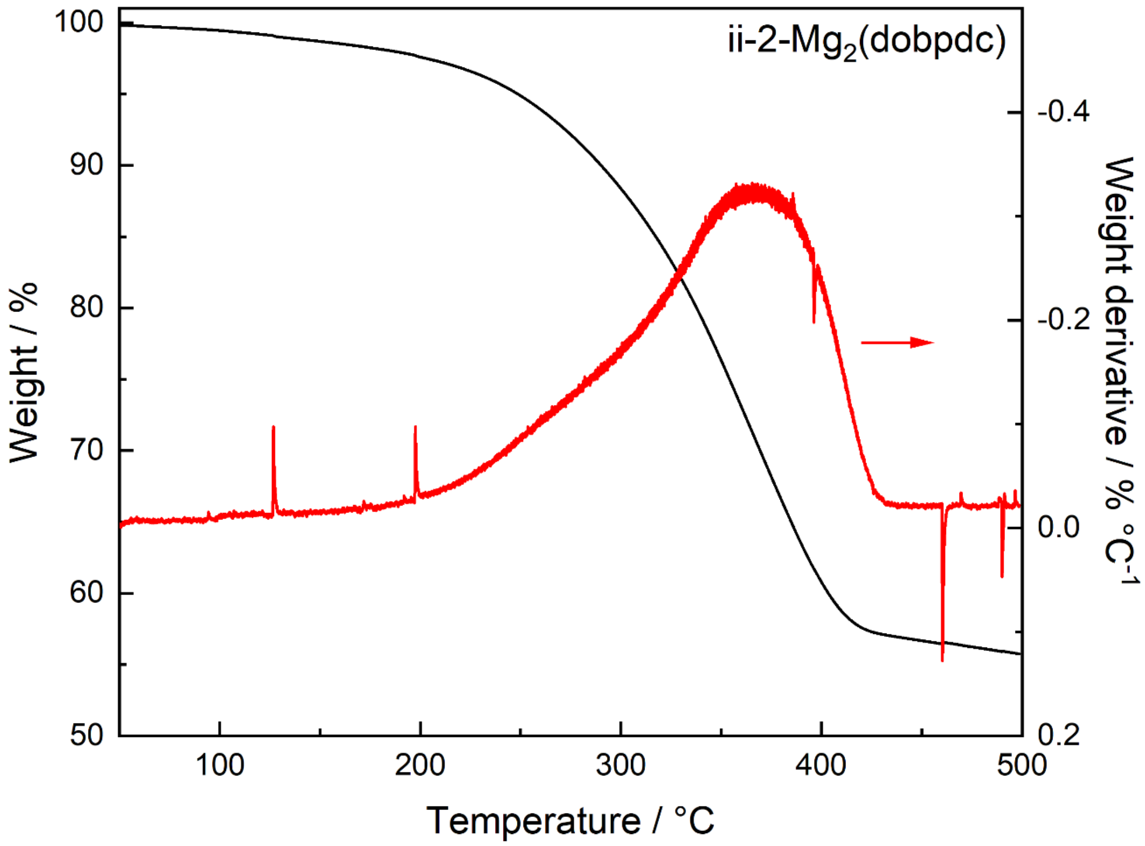

Supplementary Figure 21: TGA curve of ii-2-Mg_2(dobpdc) under dynamic heating (3 °C min^{-1}) in N_2 atmosphere.

Supplementary Notes-Gas sorption measurements

An *equilibration time* of 12 min was selected for our experiments. Hence, for Δ*t* = 12 min the pressure change should be less 0.068 mbar min^{-1}. The total amount of time elapsed for each pressure point was thus higher than 12 min. A column graph with the total time for each pressure point for CO_2 measurement of **PL7_10** at 25 °C is provided below (Supplementary Figure 22) to offer insight to the reader.

We investigated the influence of the *equilibration criterion* Δ*t* on the shape of the CO_2 sorption isotherm. Specifically, we prepared a new batch of ZIF-7, formulated a new sample of **PL7_10** and collected CO_2 isotherms at 25 °C applying an *equilibration criterion* of Δ*t* = 12 min (as in the original experiment) and also Δ*t* = 18 min in a second experiment (both done with continuous stirring). The relevant Supplementary Figures 23 and 24 are provided below. Despite the fact that a Δ*t* of 18 min resulted in longer elapsed time for each pressure point (except for two pressure points) and overall an about 50% longer data collection time (the total experiment time was 19.7 h for a Δ*t* of 12 min and 29.1 h for a Δ*t* of 18 min), the transition steps occurred at virtually the same pressures and a diminished hysteresis was not observed for a Δ*t* of 18 min.

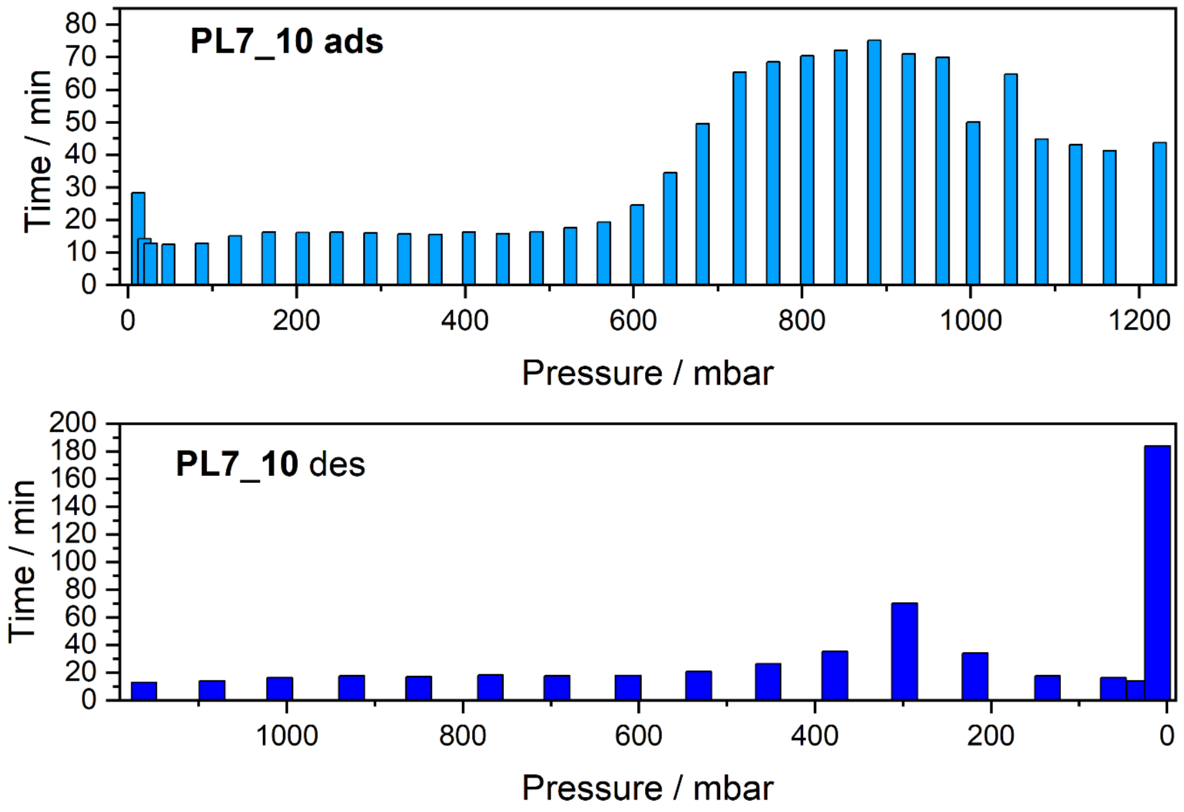

Supplementary Figure 22: Column graph with the time elapsed at each pressure point during **PL7_10** CO_2 adsorption (top) and desorption (bottom) isotherms collection at 25 °C. The data correspond to the isotherm displayed in Figure 4b.

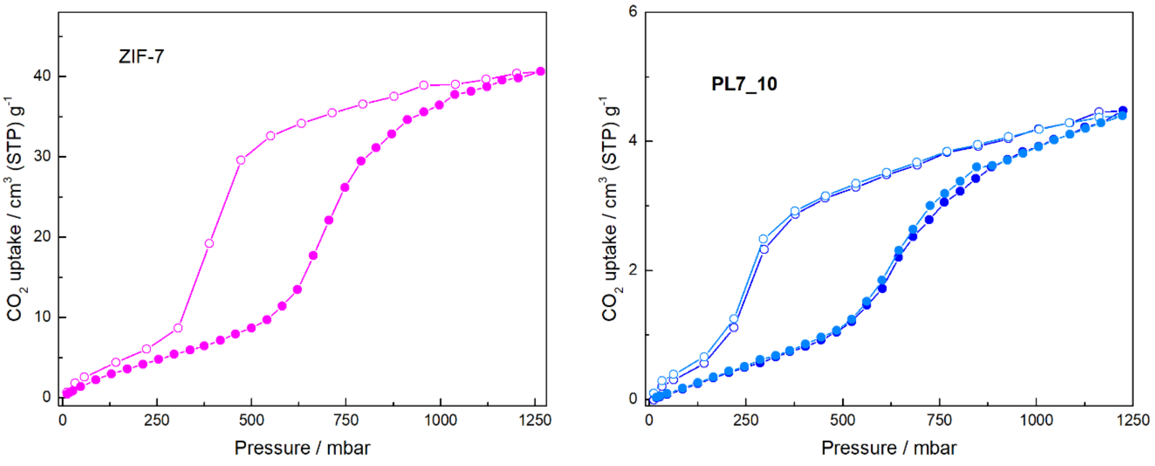

Supplementary Figure 23: Left: CO_2 sorption isotherms of ZIF-7 nanoparticles at 25 °C (left). Right: CO_2 sorption isotherms of **PL7_10** at 25 °C applying an equilibration time of 18 min (blue) and 12 min (light blue). The adsorption branch is shown with filled symbols and the desorption branch with empty symbols.

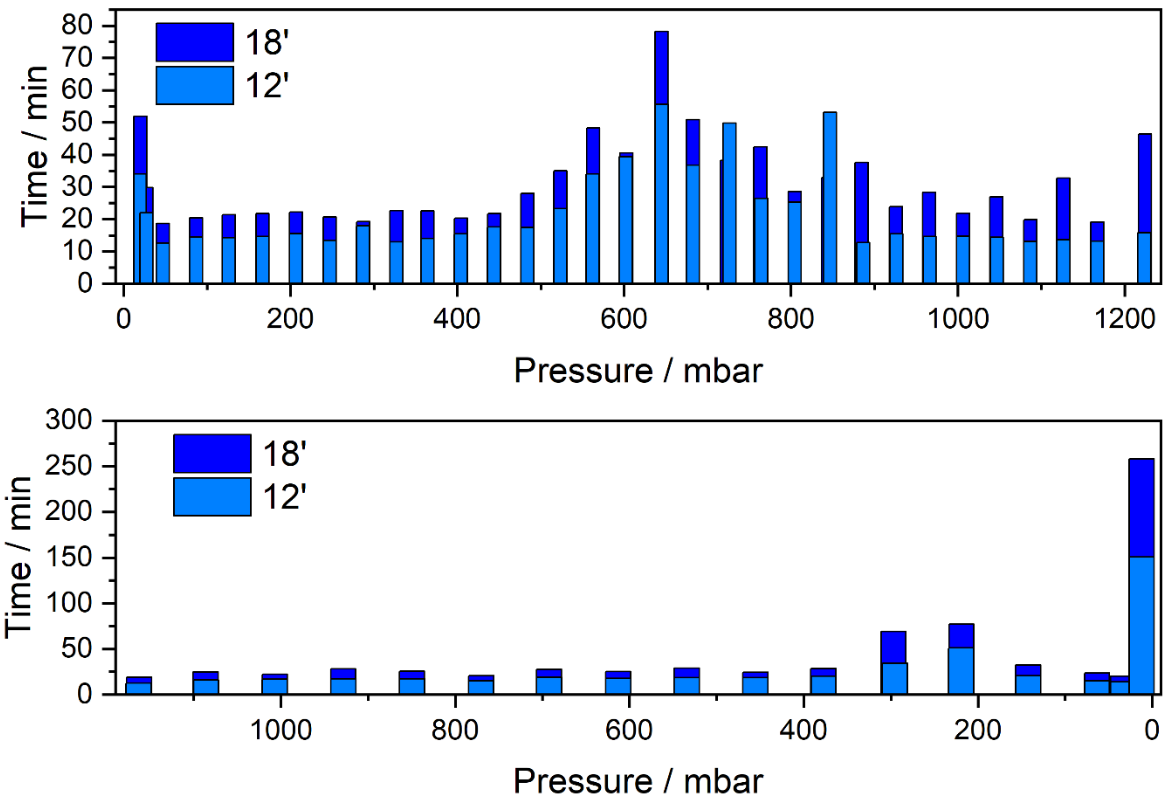

Supplementary Figure 24: Column graph with the time elapsed at each pressure point during **PL7_10** CO_2 adsorption (top) and desorption (bottom) isotherms collection at 25 °C applying a Δt of 18 min (blue) and 12 min (light blue). The total experiment time has been 29.1h for Δt = 18 min and 19.7 h for Δt = 12 min.

The ideal uptakes of **PL7_10** and **PL7_5**, based on the weighted contributions of their constituents, were calculated according to the following equation: $n_{\mathrm{PL_{id}}} = (\omega_{\mathrm{S}} \cdot n_{\mathrm{S}}) + (\omega_{\mathrm{ZIF}} \cdot n_{\mathrm{ZIF}})$, where ω_{ZIF}

and ω_S refer to the mass ratio of ZIF-7 and Silicone 704 in the formulated PLs, and n_S and n_{ZIF} denote the CO_2 uptake per mass unit of Silicone 704 and ZIF-7. The CO_2 uptake of Silicone 704 at the investigated pressures was calculated based on the fit of its experimental isotherm that is displayed below.

The uptakes of **PL7_10** and **PL7_5** normalized per unit mass of ZIF-7 (Figure 2d) were calculated according to the following equation: $n_{PL/g_{ZIF}} = \frac{n_{PL} - \omega_S\, n_S}{\omega_{ZIF}}$, where n_{PL} is the experimental CO_2 uptake of the PL. The CO_2 uptake of Silicone 704 at the investigated pressures was calculated based on the fit of its experimental isotherm that is displayed below.

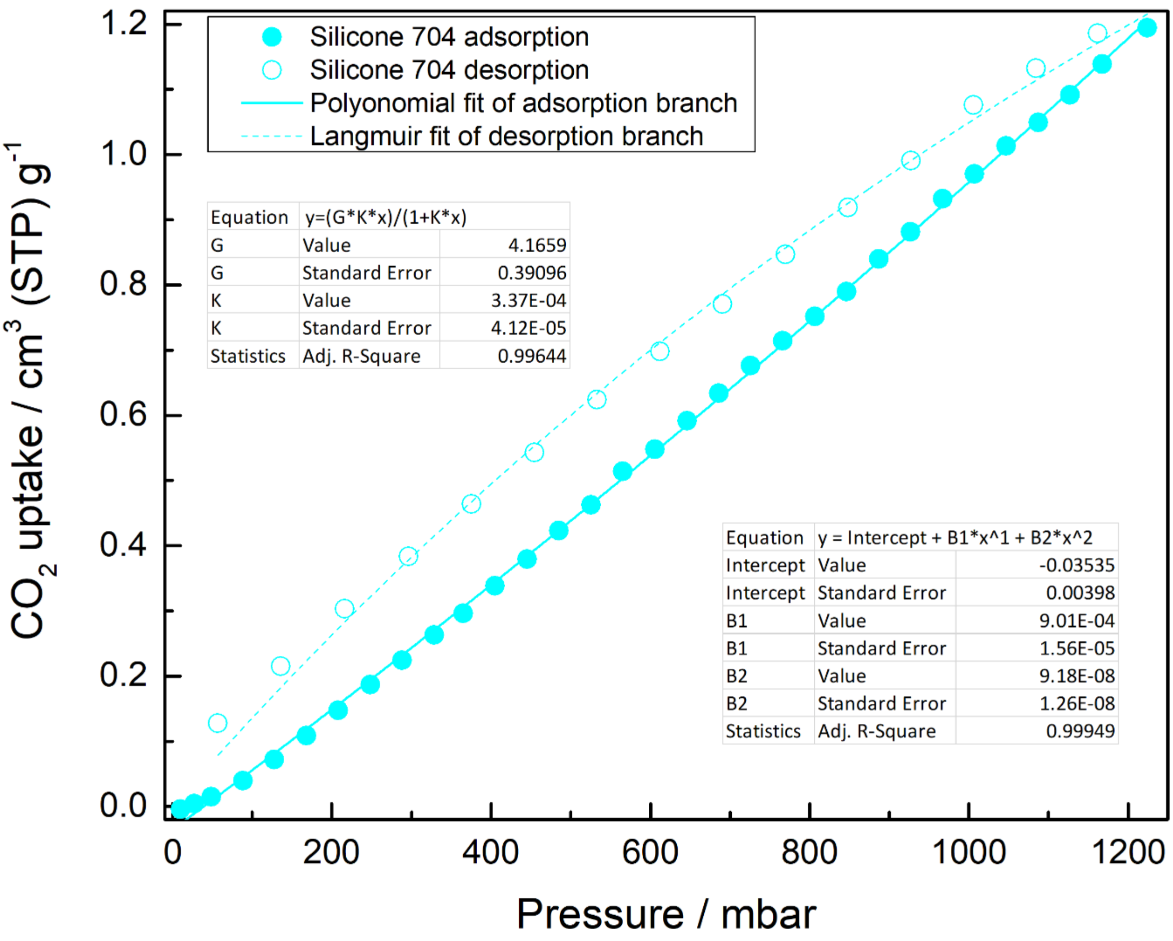

Supplementary Figure 25: Fits of the experimentally measured CO_2 isotherms by Silicone 704 at 25 °C. Solid and dashed lines display the fits of the adsorption and the desorption branch, respectively. Tables summarizing the parameters of the fits are provided.

A linear isotherm for CO_2 sorption in silicone 704 would be expected. It appears though that the absorption of CO_2 is kinetically hindered, so that the isotherm features a slight curvature and a slight hysteresis between absorption and desorption (Supplementary Figure 23) indicating that the isotherm does not represent equilibrium conditions.

In order to investigate if equilibrium can be reached by longer equilibration times, we then collected additional CO_2 sorption isotherms of Silicone 704 using larger Δt values („*equilibration times*“, see

above) of 16 and 24 mins (instead of 12 min used previously). The results are provided in Supplementary Figure 24. It appears that longer Δt leads to a small increase in the overall CO_2 uptake at ca. 1200 mbar. The width of the hysteresis between absorption and desorption, however, is largely unaffected by the longer Δt. This experiment established that CO_2 sorption in Silicone 704 is indeed kinetically hindered, even though the liquid is continuously stirred during data collection.

Linear fits to the sorption isotherms are not satisfactory because of the slight curvature of the isotherms and the hystereses. Thus, we applied polynomial (absorption) and Langmuir (desorption) functions to derive much better fits to the experimental data.

We used the isotherm collected with a Δt of 12 min for the comparison of the sorption behaviour of silicone 704 with the PLs reported here, since the data of the PLs have also been collected with the same Δt setting. One might argue that the isotherms of pure silicone 704 are not fully equilibrated when the rather short Δt of 12 min is applied, however, a reference experiment of PL7_10 with an increased Δt (18 min instead of 12 min, see Supplementary Figures 21 and 22) shows that the increased Δt leads to insignificant changes in the isotherm shape (i.e. phase transition pressures and hysteresis width) and overall gas uptake of the PL. Thus, all the reported gas sorption isotherms of the PLs in this work must be regarded as reasonably well equilibrated.

We may note again that breathing MOFs typically show step-shaped gas sorption with a rather strong hysteretic behaviour. The hysteresis between ad- and desorption is a consequence of the activation energy of the phase transitions (i.e. it is a kinetic effect) and real thermodynamic equilibrium is not achievable when recording sorption isotherms of such systems with experimental time scales.[4] In other words, the phase transition region of an experimental sorption isotherm of a breathing MOF is never a representation of thermodynamic equilibrium. Naturally the same is true for the breathing PLs discussed in this work.

Supplementary Figures-Gas sorption measurements

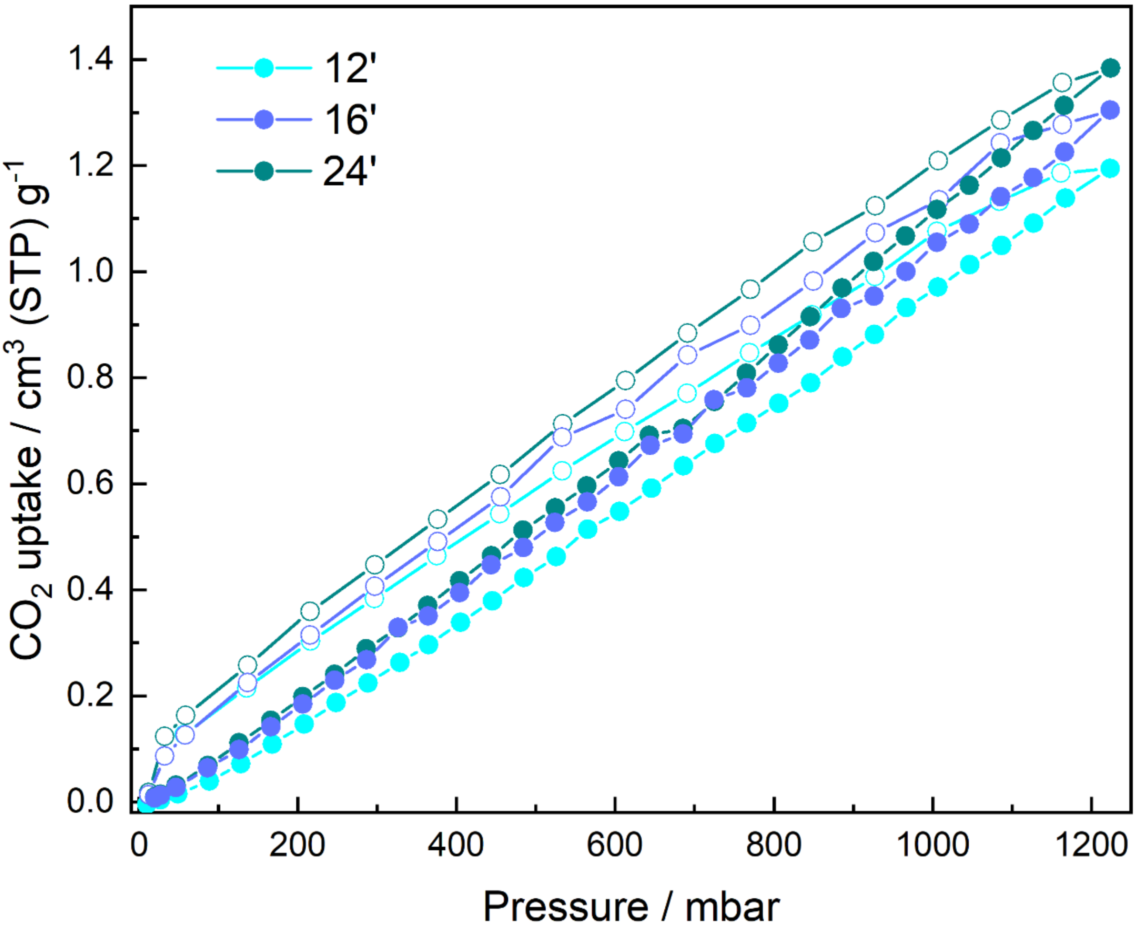

Supplementary Figure 26: Silicone 704 CO_2 isotherms at 25 °C recorded with a Δt setting of 12 min (cyan), 16 min (purple) and 24 min (dark green).

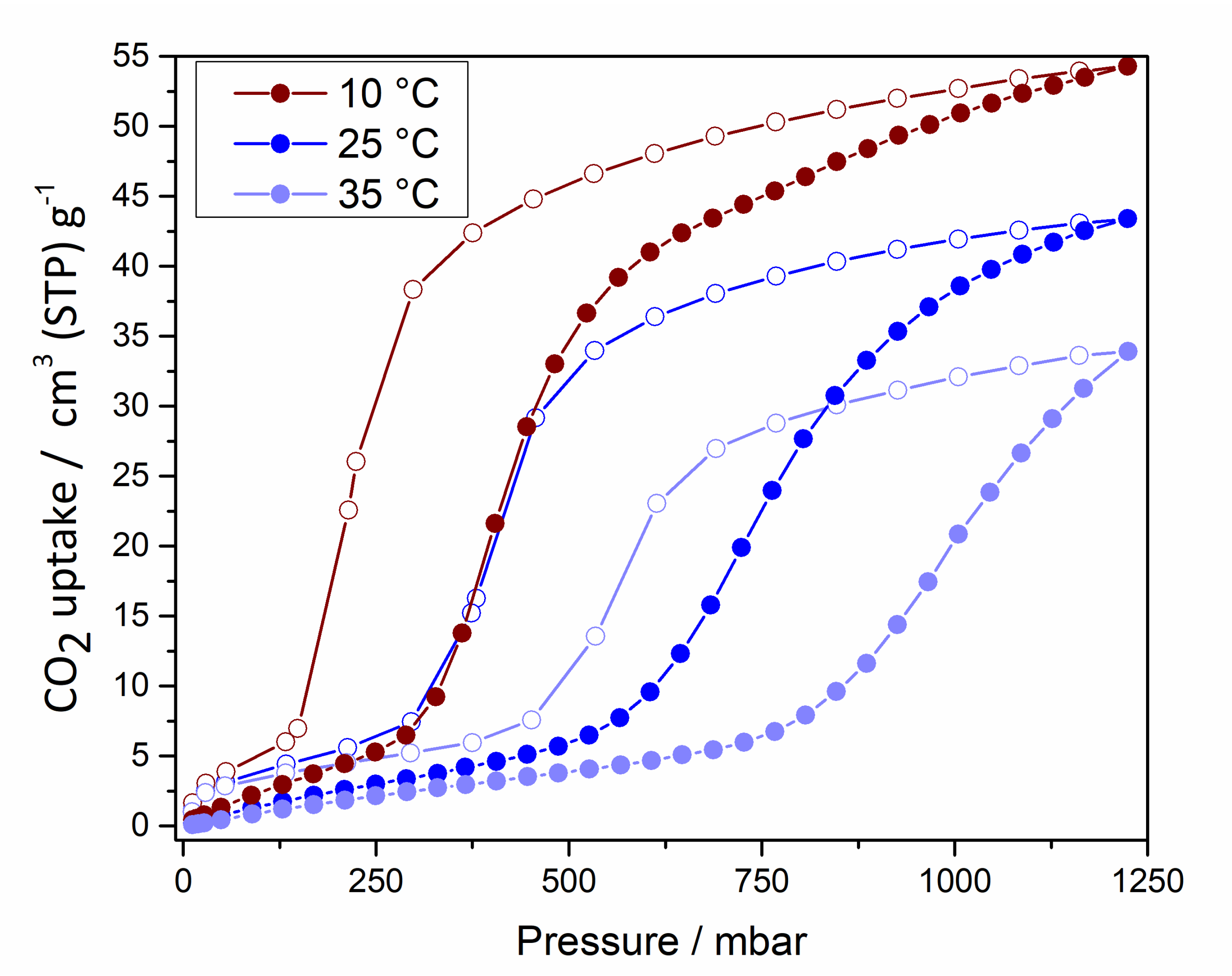

Supplementary Figure 27: CO_2 sorption isotherm for ZIF-7 nanocrystals at 10 °C (burgundy), 25 °C (blue) and 35 °C (light purple). For all isotherms the adsorption branch is shown with filled symbols and the desorption branch with empty symbols.

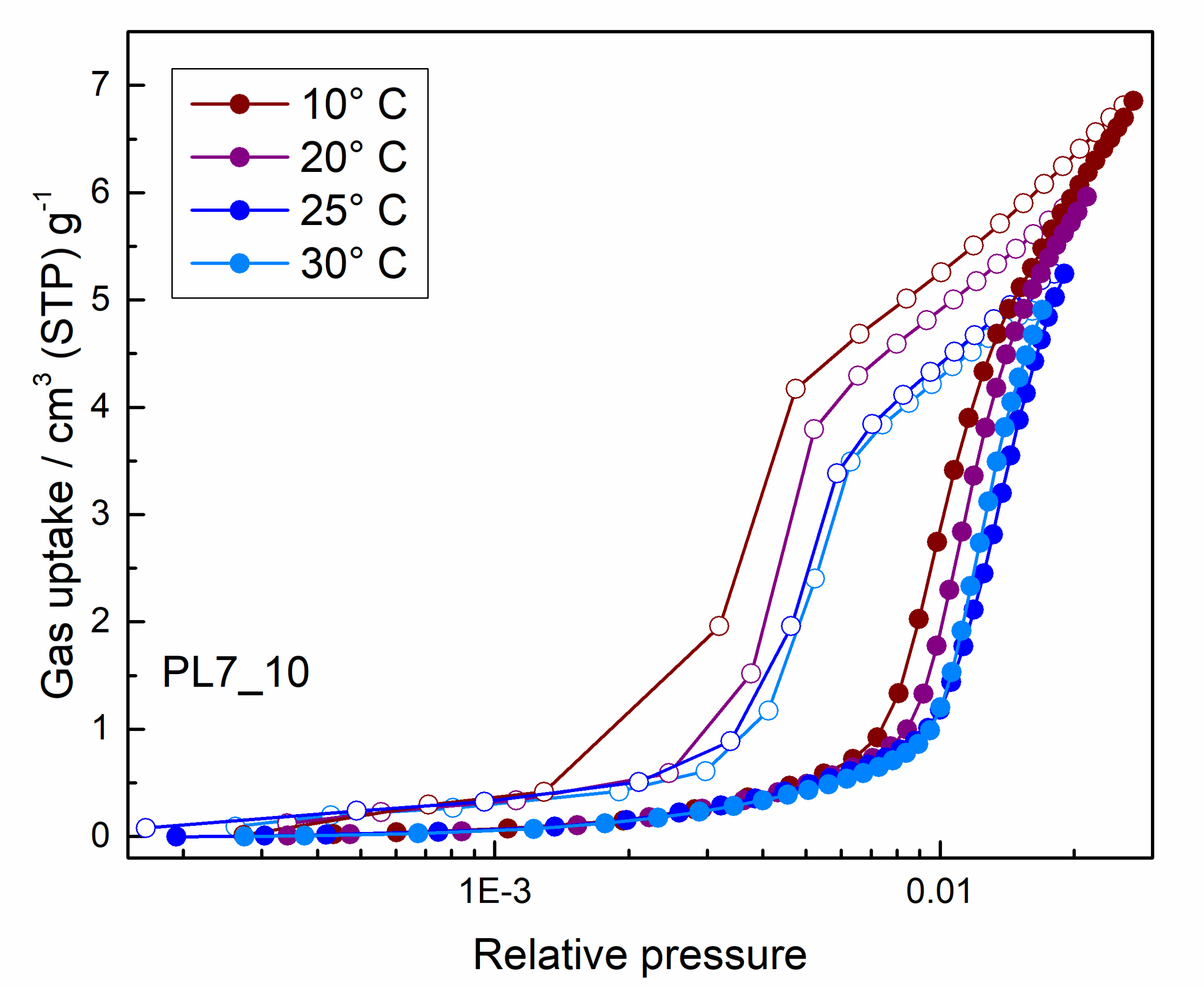

Supplementary Figure 28: CO_2 sorption isotherms for **PL7_10**, filled symbols denote the adsorption branch and empty symbols denote the desorption branch, collected at 10, 20, 25 and 30 °C. The logarithmic horizontal axis is plotted in relative pressure (p/p_0).[5] The isotherm for **PL7_10** recorded at 35 °C was not included because 35 °C is above the critical temperature of CO_2, so that the saturation pressure p_0 is not defined.

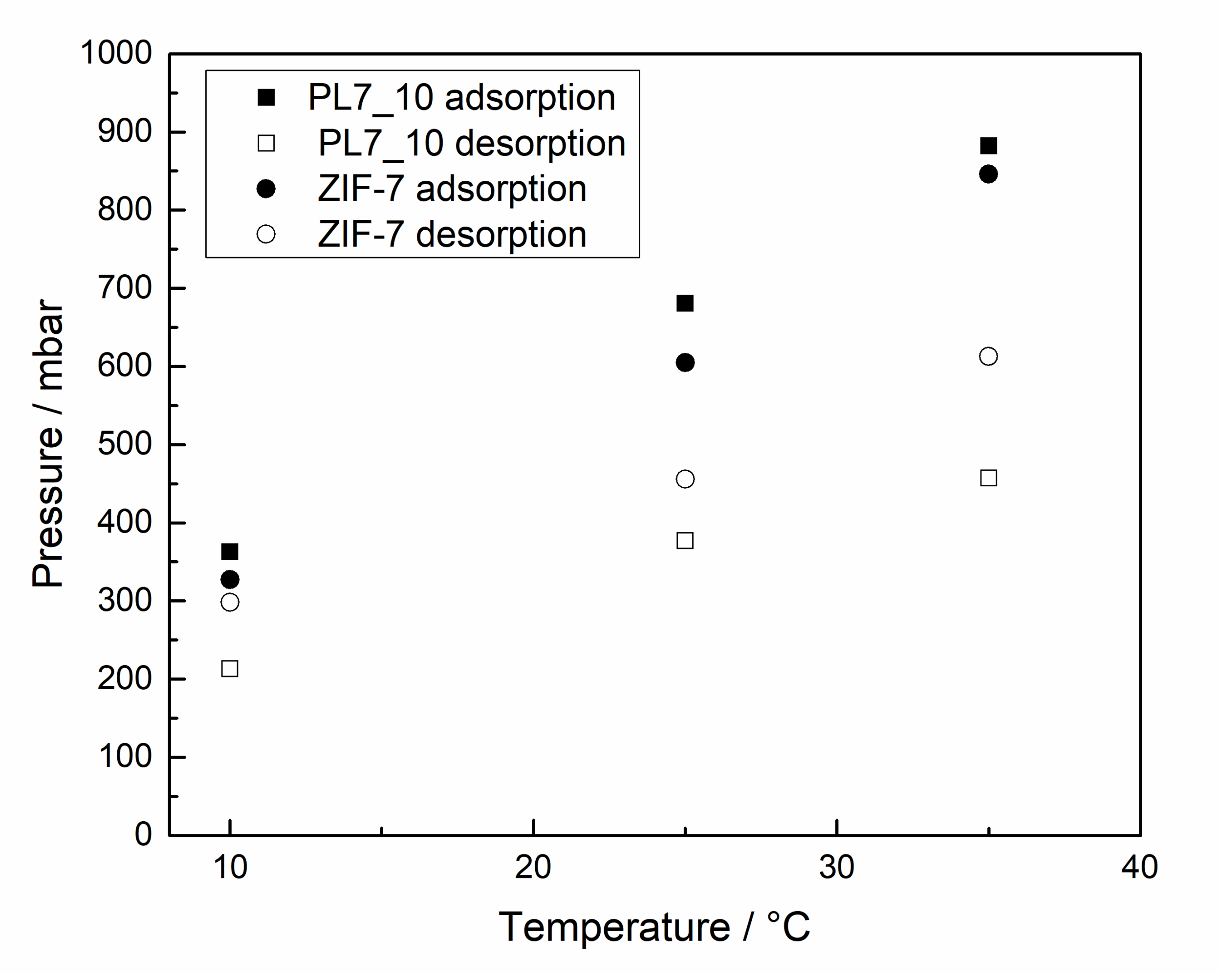

Supplementary Figure 29: Phase transition pressures measured in adsorption (filled symbols) and desorption (empty symbols) isotherms for ZIF-7 (circles) and **PL7_10** (squares) as a function of temperature. The transition pressures are identified at the onset of the gate opening (or gate closing for desorption) in the isotherms.

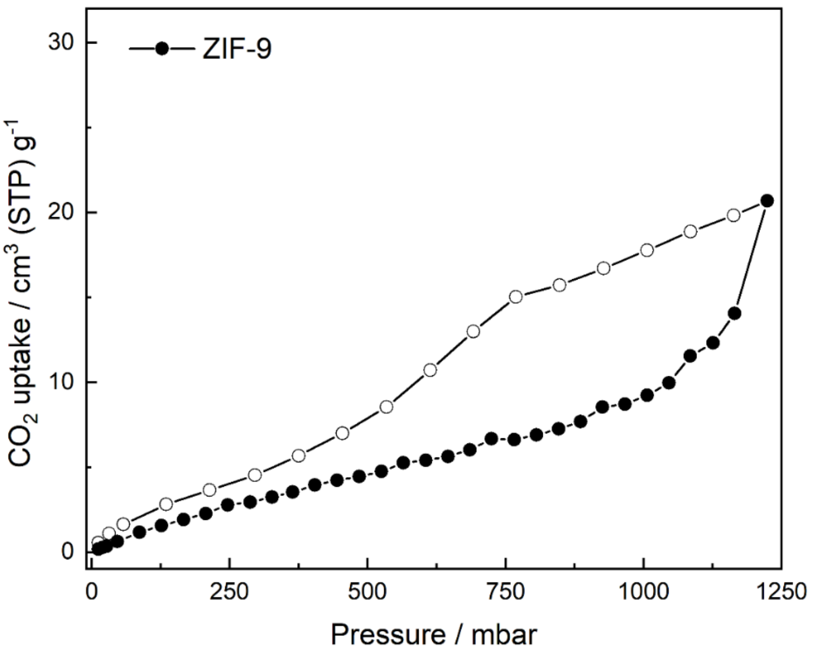

Supplementary Figure 30: CO_2 sorption isotherms for ZIF-9 collected at 25 °C. Filled symbols denote the adsorption branch and empty symbols denote the desorption branch. The phase transition to the ***lp*** phase is not yet completed at a pressure of 1224 mbar (the maximum gas pressure of the instrument used).

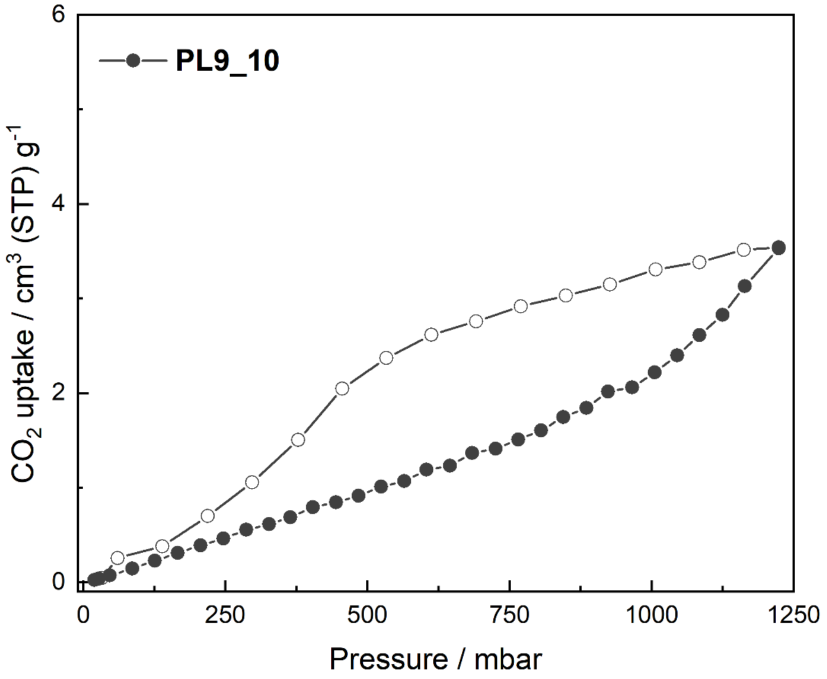

Supplementary Figure 31: CO_2 sorption isotherms for **PL9_10** collected at 25 °C. Filled symbols denote the adsorption branch and empty symbols denote the desorption branch. The phase transition of ZIF-9 to the ***lp*** phase is not yet completed at a pressure of 1225 mbar (the maximum gas pressure of the instrument used).

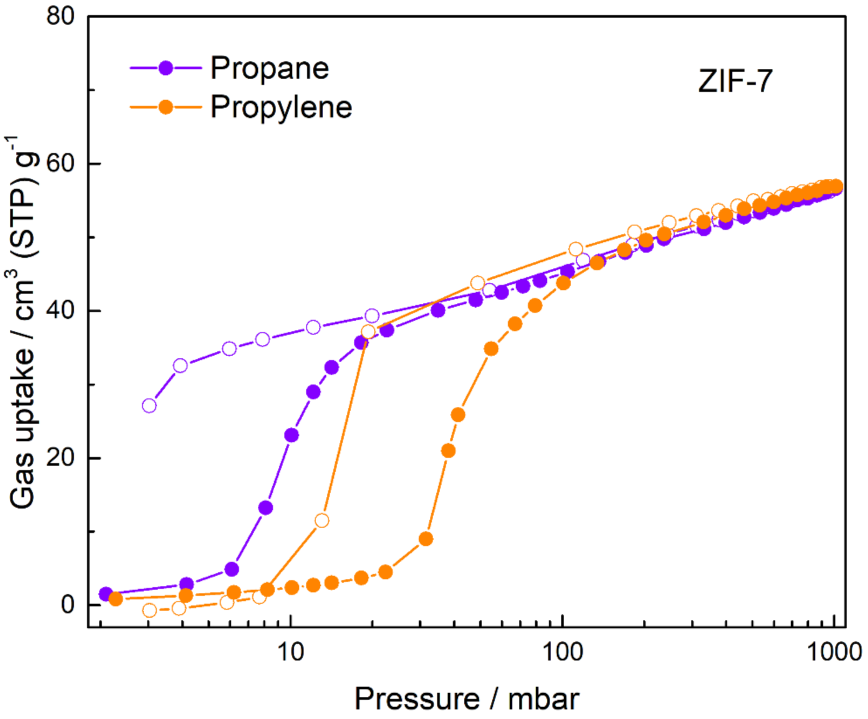

Supplementary Figure 32: Propane (purple) and propylene (orange) sorption isotherms for ZIF-7 collected at 25 °C. Filled symbols denote the adsorption branch and empty symbols denote the desorption branch.

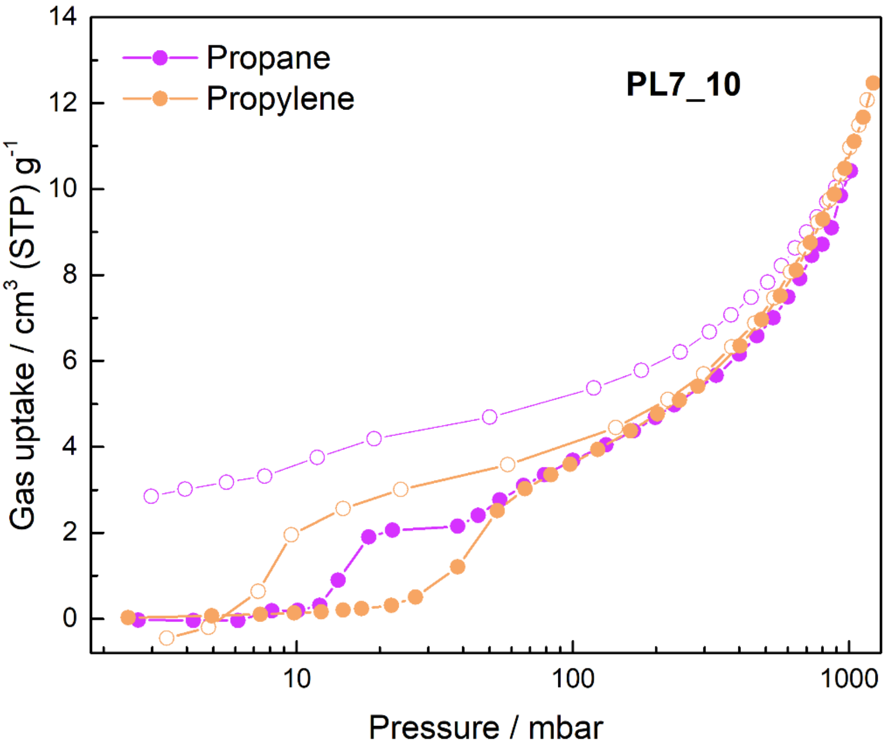

Supplementary Figure 33: Propane (light purple) and propylene (light orange) sorption isotherms for **PL7_10** collected at 25 °C. Filled symbols denote the adsorption branch and empty symbols denote the desorption branch.

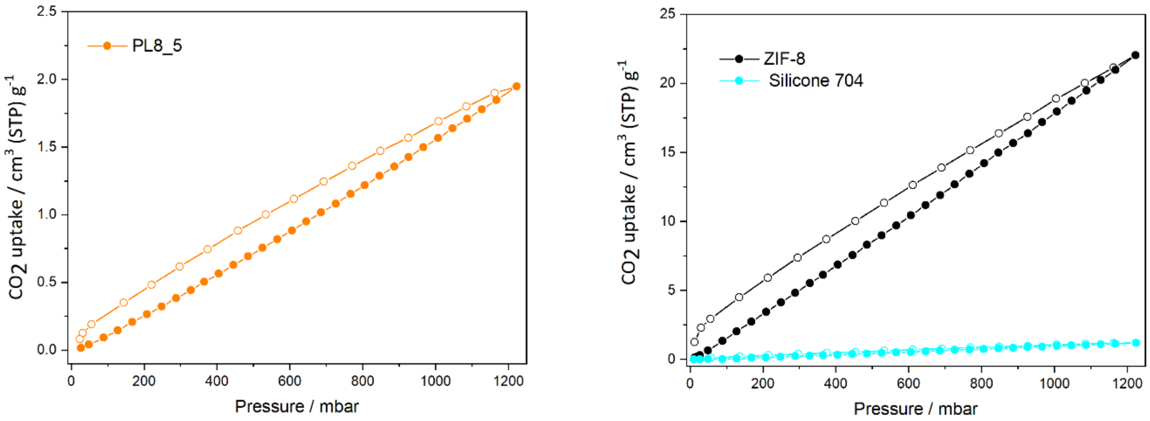

Supplementary Figure 34: CO_2 sorption isotherm of **PL8_5** (left) and its constituents, ZIF-8 nanocrystals and Silicone 704 (right), at 25 °C. For all isotherms the adsorption branch is shown with filled symbols and the desorption branch with empty symbols.

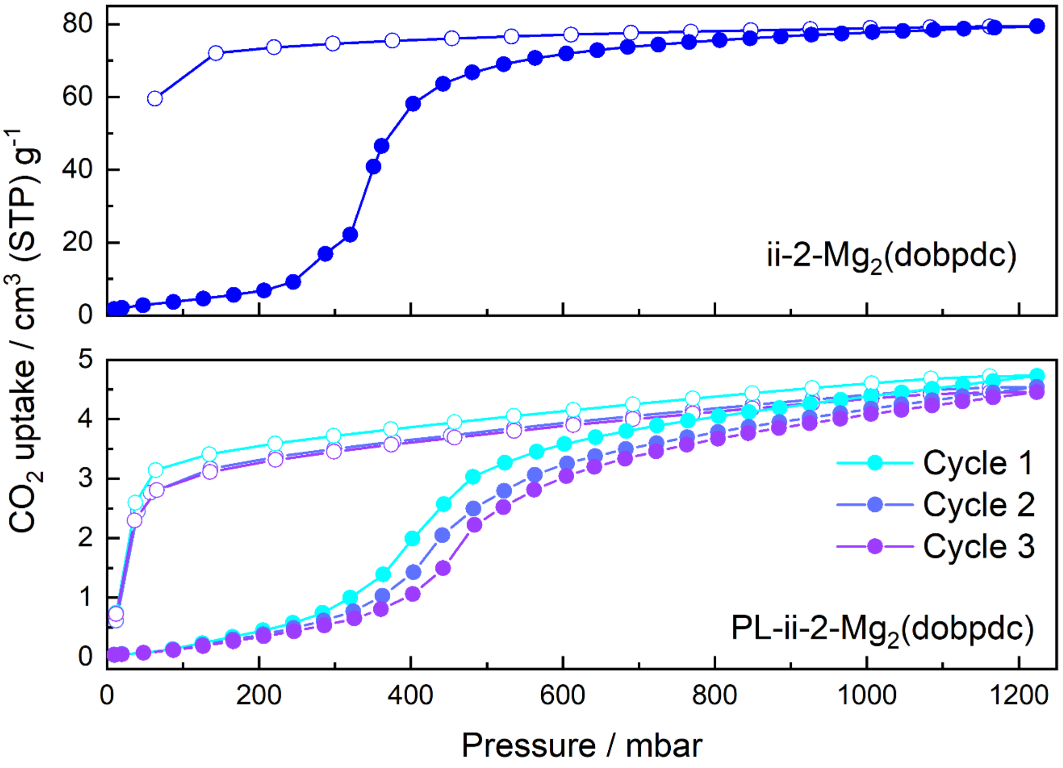

Supplementary Figure 35: CO_2 sorption isotherms of ii-2-Mg_2(dobpdc) (top) and its respective 5wt% PL in Silicone 704 (bottom) at 25 °C. For all isotherms the adsorption branch is shown with filled symbols and the desorption branch with empty symbols, while the lines are a guide to the eye. Similar to the observations for the **PL7** and **PL9** materials, the step during adsorption is shifted to slightly higher gas pressures, whereas the step during desorption is shifted to slightly lower gas pressures. The uptake of the PL at 1224 mbar is in good agreement with the uptake expected from the constituents, with the experimental value being equal to 93% of the ideal uptake. It is estimated that the Silicone 704 contributes a CO_2 uptake of about 1.13 cm^3 (STP) g^{-1} PL, while the ii-2-Mg_2(dobpdc) particles contribute about 3.60 cm^3 (STP) g^{-1} PL. This is quite impressive, since the 5 wt% ii-2-Mg_2(dobpdc) particles in the PL contribute about 76% of the CO_2 uptake capacity of the PL at 1224 mbar. Three adsorption/desorption cycles were carried out with the PL being regenerated under vacuum at 100 °C and 120 °C, before cycle 2 and cycle 3, respectively. Between cycles 1 and 2 the sample rested for 4 days at ambient conditions.

Supplementary Figures-In situ XRD

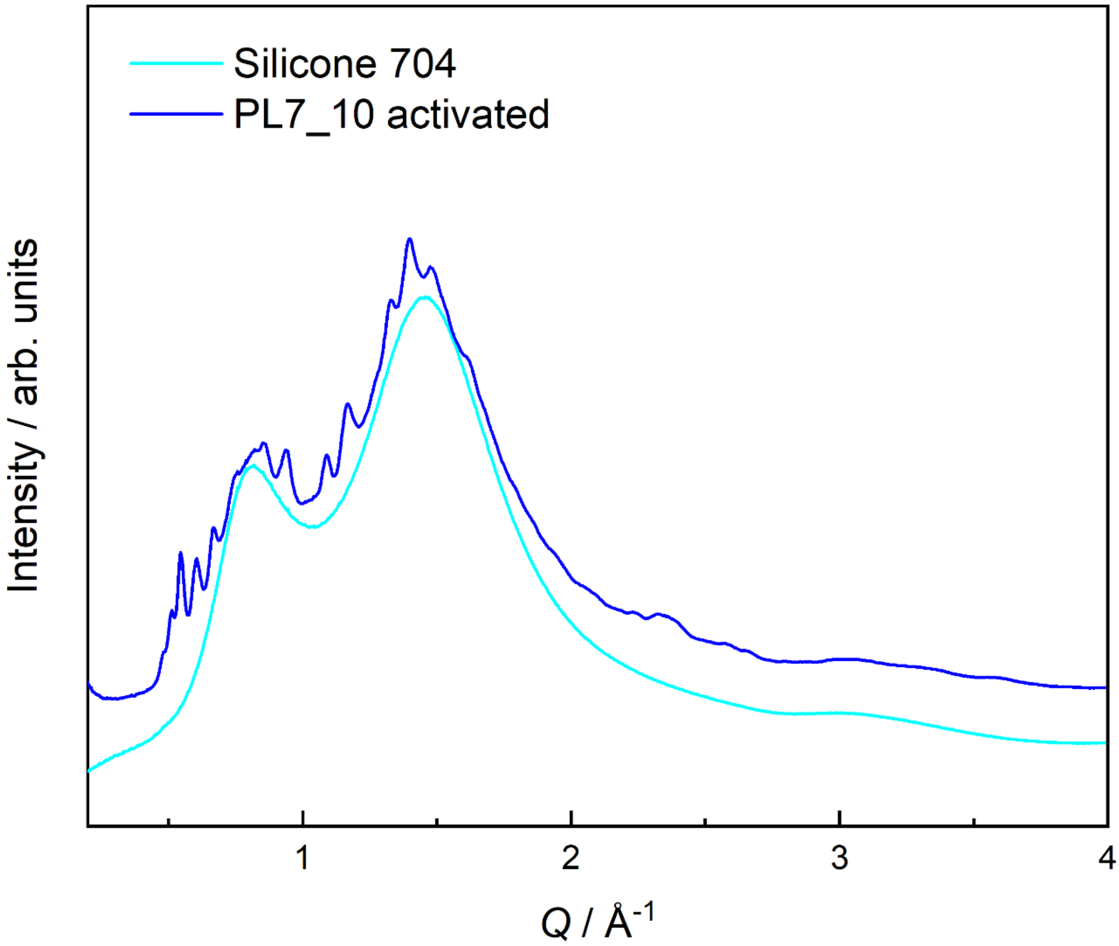

Supplementary Figure 36: XRD pattern of neat Silicone 704 in comparison to the pattern of activated **PL7_10**.

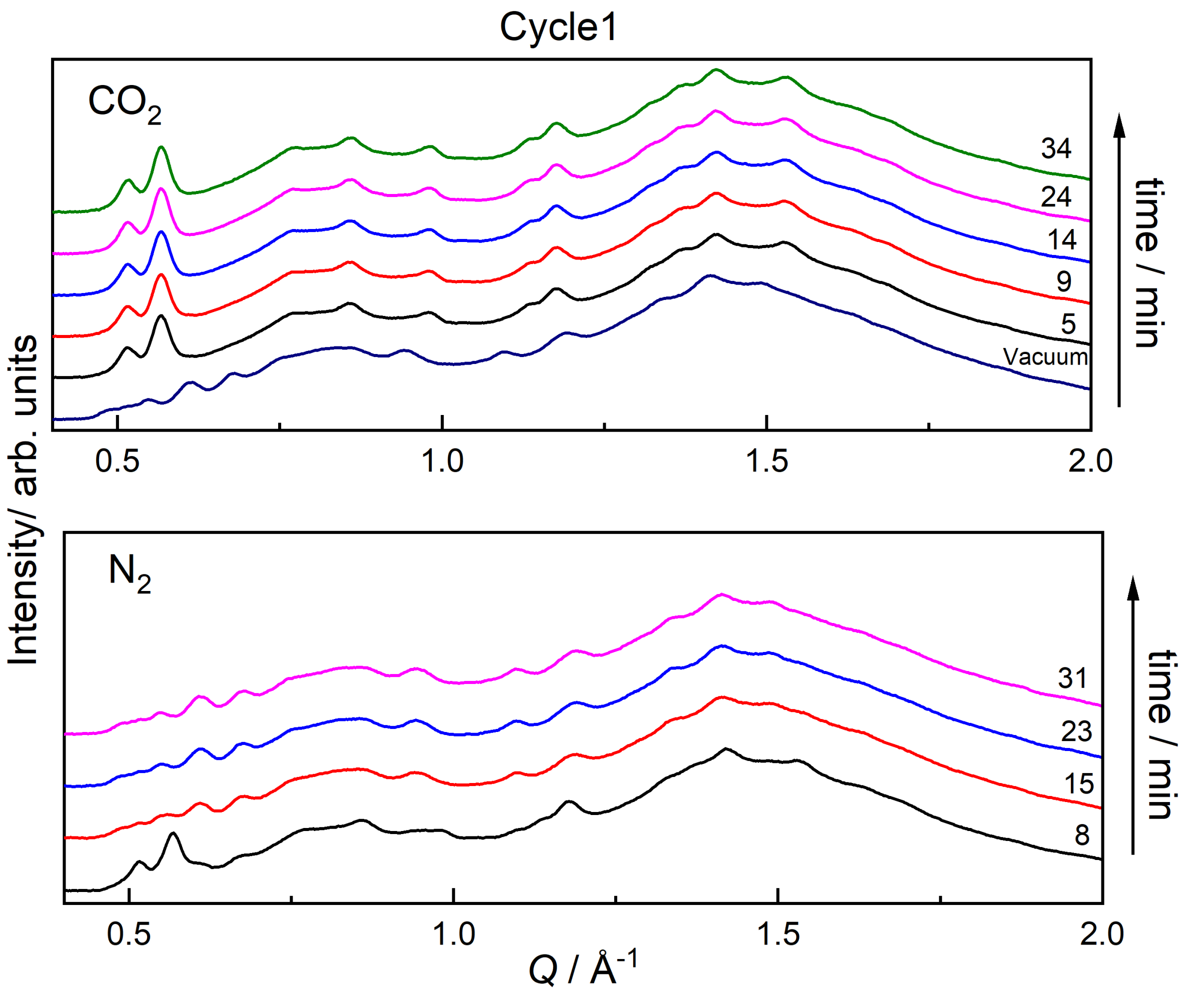

Supplementary Figure 37: Time-resolved *in situ* XRD patterns during gas uptake by **PL7_10** (cycle 1). Exposure time and detector readout time sum up to about 4 – 5 min. So that diffraction patterns could only be measured in time intervals of about 4 – 5 min. Top: Sequential XRD patterns recorded during bubbling CO_2 through **PL7_10**. Bottom: Sequential XRD patterns recorded during bubbling N_2 through **PL7_10**.

.

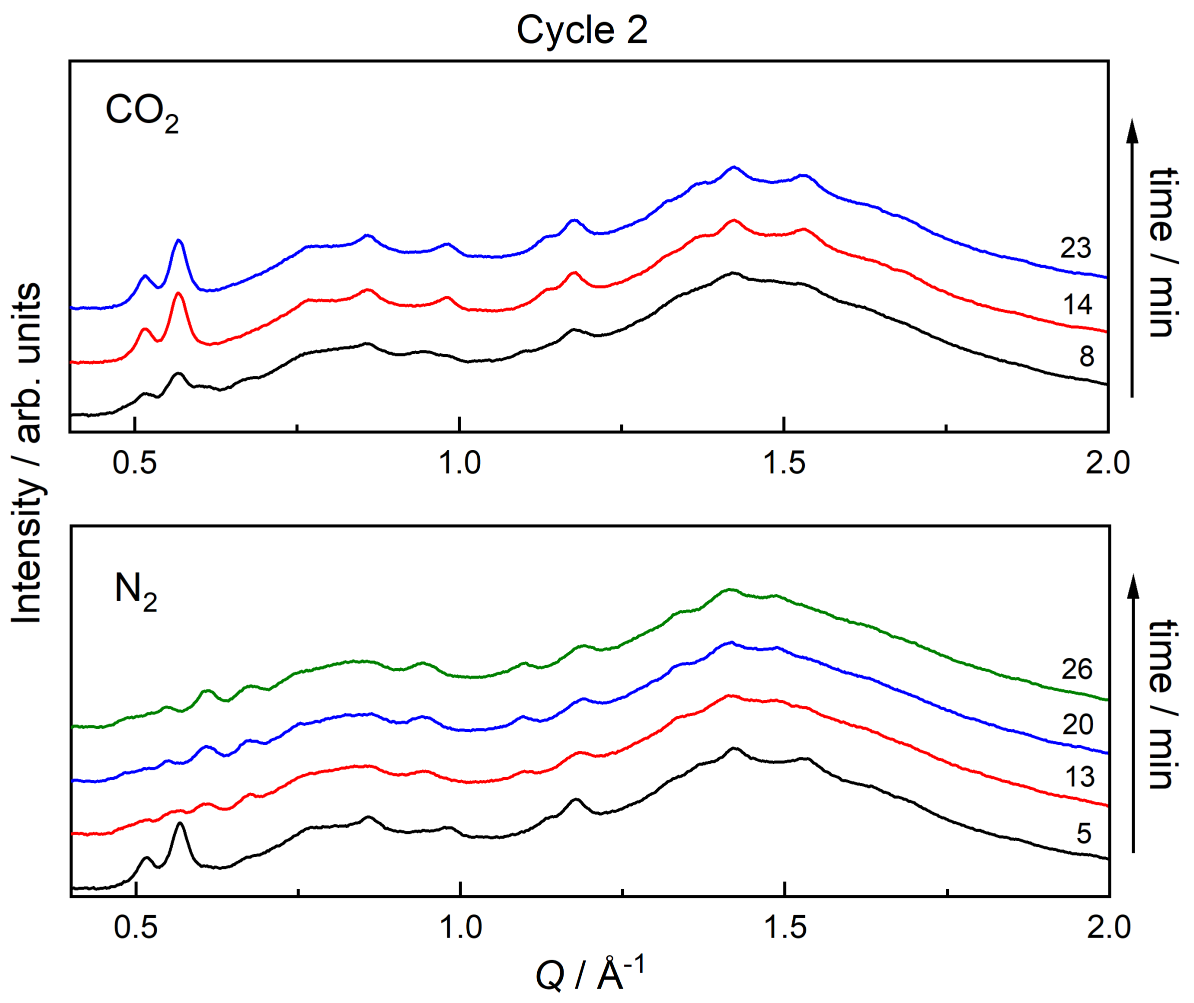

Supplementary Figure 38: Time-resolved *in situ* XRD patterns during gas uptake by **PL7_10** (cycle 2). Exposure time and detector readout time sum up to about 4 – 5 min. So that diffraction patterns could only be measured in time intervals of about 4 – 5 min. Top: Sequential XRD patterns recorded during bubbling CO_2 through **PL7_10**. Bottom: Sequential XRD patterns recorded during bubbling N_2 through **PL7_10**.

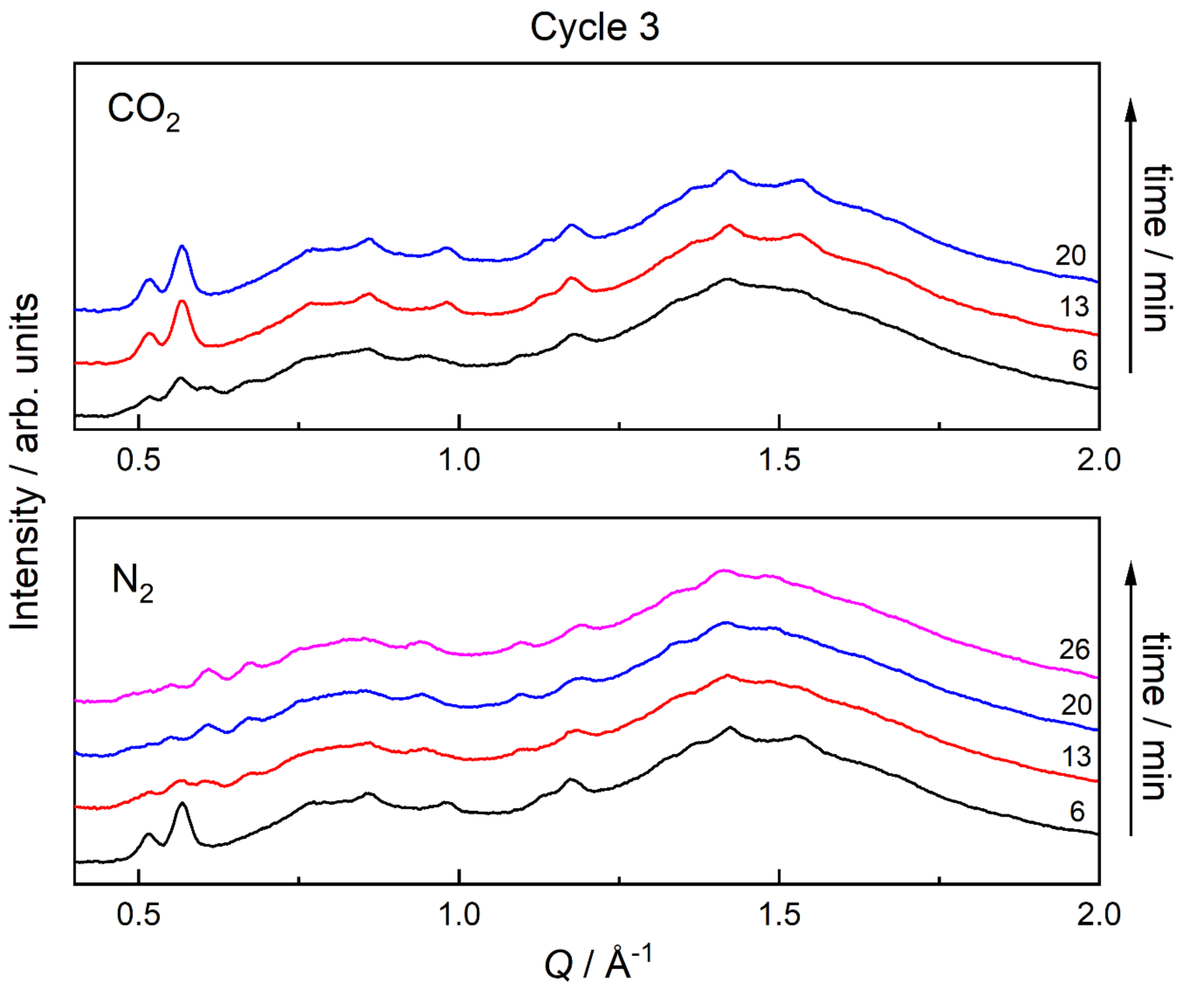

Supplementary Figure 39: Time-resolved in situ XRD patterns during gas uptake by **PL7_10** (cycle 3). Exposure time and detector readout time sum up to about 4 – 5 min. So that diffraction patterns could only be measured in time intervals of about 4 – 5 min. Top: Sequential XRD patterns recorded during bubbling CO_2 through **PL7_10**. Bottom: Sequential XRD patterns recorded during bubbling N_2 through **PL7_10.**

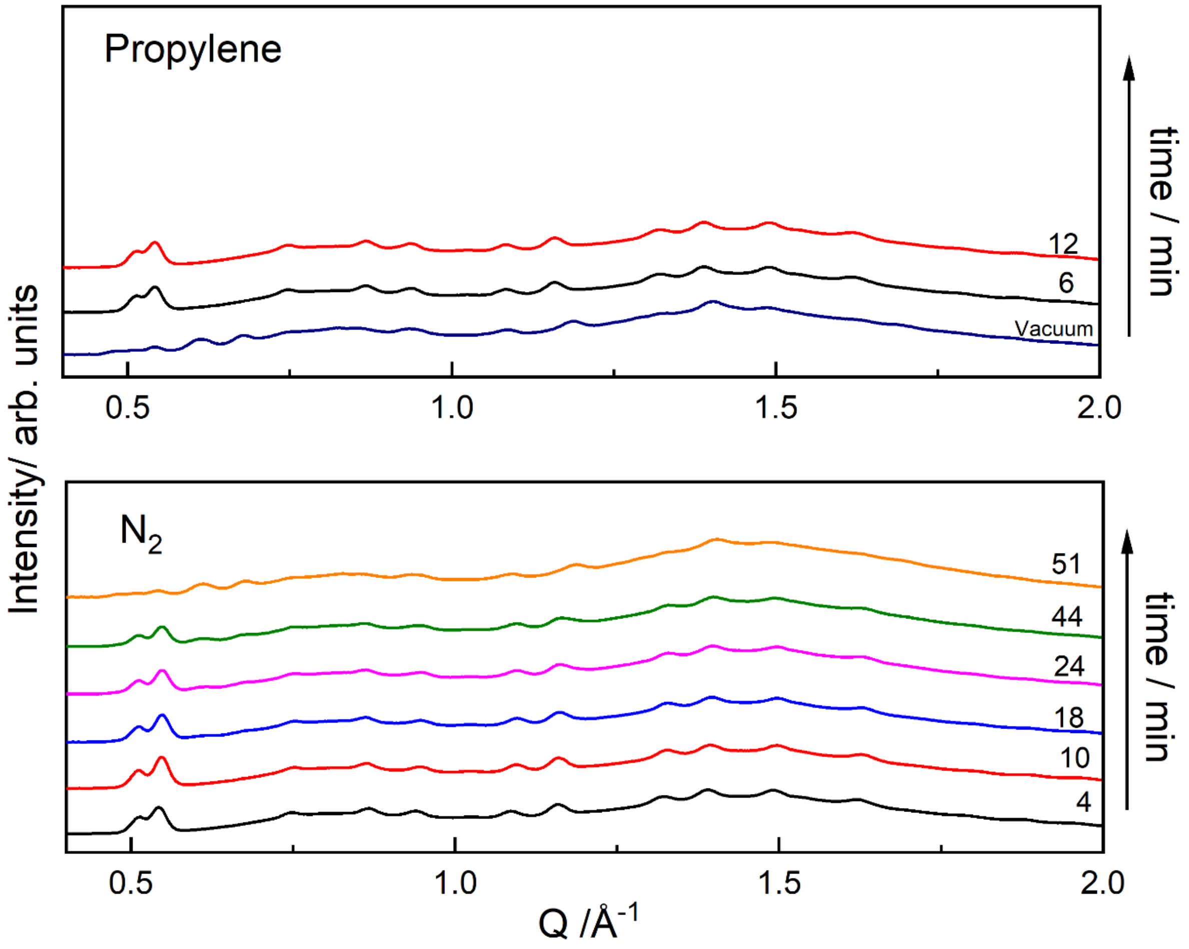

Supplementary Figure 40: Time-resolved *in situ* XRD patterns during gas uptake by **PL9_10**. Exposure time and detector readout time sum up to about 4 – 5 min. So that diffraction patterns could only be measured in time intervals of about 4 – 5 min. Top: Sequential XRD patterns recorded during bubbling propylene through **PL9_10**. Bottom: Sequential XRD patterns recorded during bubbling N_2 through **PL9_10**.

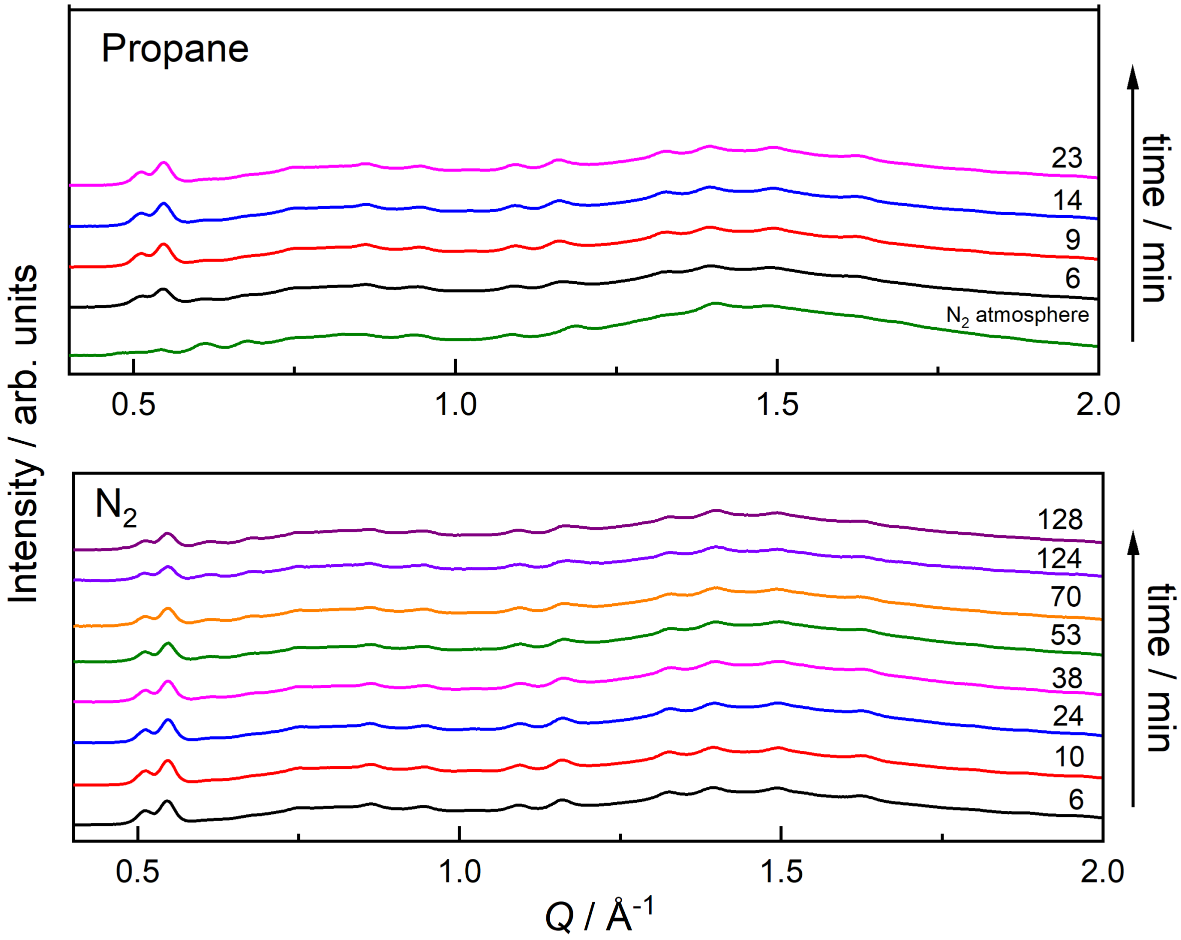

Supplementary Figure 41: Time-resolved in situ XRD patterns during gas uptake by **PL9_10**. Exposure time and detector readout time sum up to about 4 – 5 min. So that diffraction patterns could only be measured in time intervals of about 4 – 5 min. Top: Sequential XRD patterns recorded during bubbling propane through **PL7_10**. Bottom: Sequential XRD patterns recorded during bubbling N_2 through **PL7_10**.

Supplementary Tables

Supplementary Table 1: Unit cell parameters and corresponding R_{wp}, R_{exp} and GOF values determined by the above displayed structureless profile fits (Pawley method).

material	ZIF-7 *np*	ZIF-7 *lp*	ZIF-9 *np*	ZIF-9 *lp*	ZIF-8	PL7_10 CO_2 saturated
**crystal system**	triclinic	trigonal	triclinic	trigonal	cubic	trigonal
**space group**	$P\bar{1}$	$R\bar{3}$	$P\bar{1}$	$R\bar{3}$	$I\bar{4}3m$	$R\bar{3}$
$\boldsymbol{a}$ **/ Å**	11.494(5)	23.066(6)	11.336(8)	23.093(3)	17.059(1)	22.234(6)
$\boldsymbol{b}$ **/ Å**	14.340(9)	23.066(6)	14.282(14)	23.093(3)	17.059(1)	22.234(6)
$\boldsymbol{c}$ **/ Å**	14.423(8)	15.786(5)	14.378(12)	15.800(2)	17.059(1)	15.836(5)
$\boldsymbol{\alpha}$ **/ °**	109.94(2)	90	110.09(4)	90	90	90
$\boldsymbol{\beta}$ **/ °**	95.790(15)	90	95.50(2)	90	90	90
$\boldsymbol{\gamma}$ **/ °**	109.645(19)	120	109.31(3)	120	90	120
$\boldsymbol{V}$ **/ Å**^{3}	2041(2)	7273(5)	2005(3)	7297(2)	4964.7(12)	6780(4)
$\boldsymbol{R_{wp}}$ **/ %**	4.525	18.141	7.215	37.671	18.592	0.386
$\boldsymbol{R_{exp}}$ **/ %**	2.851	16.257	5.684	31.798	15.033	4.037
**GOF**	2.519	1.245	1.611	1.404	1.530	0.100

Supplementary Table 2: CO_2 uptake values at ambient/sub-ambient conditions by PLs reported in the literature in comparison to the values recorded for the PLs investigated in this work (highlighted in blue colour).

PL	Solid phase (wt%)	Liquid phase	Pressure / bar	Temperature / °C	CO_2 uptake / cm^3 (STP g^{-1})
**zeo-PL-1[6]**	zeolite rho (12.5)	Genosorb 1753	0.8	25	10.64
**zeo-PL-2[6]**	zeolite rho (25)	Genosorb 1753	0.8	25	16.53
**Po($IL1_{0.25}IL2_{0.75}$)-Z5[7]**	ZIF-8 (5)	[$P_{6,6,6,14}$][NTf_2] and [$P_{4,4,4,4}$][OAc]	1	30	18.14
**PoIL3-Z5**	ZIF-8 (5)	[$P_{4,4,4,4}$][Lev]	1	30	25.51
**12.5 wt% CC3-*R*/CC3-*S* in Silicone oil[8]**	CC3-R/CC3-S (12.5)	Silicone oil	1	25	4.44
**20 wt% CC3-*R*/CC3-*S* in Silicone oil[8]**	CC3-R/CC3-S (20)	Silicone oil	1	25	6.27
**25 wt% CC3-*R*/CC3-*S* in Silicone oil[8]**	CC3-R/CC3-S (25)	Silicone oil	1	25	8.08
**30 wt% CC3-*R*/CC3-*S* in Silicone oil[8]**	CC3-R/CC3-S (30)	Silicone oil	1	25	8.93
**50 wt% CC3-*R*/CC3-*S* in Silicone oil[8]**	CC3-R/CC3-S (50)	Silicone oil	1	25	14.23
**12.5 wt% CC3-*R*/CC3-*S* in [BPy][NTf_2]**	CC3-R/CC3-S (12.5)	[BPy][NTf_2]	1	25	3.86
**20 wt% CC3-*R*/CC3-*S* in [BPy][NTf_2]**	CC3-R/CC3-S (20)	[BPy][NTf_2]	1	25	4.70
**PL 20 wt%[9]**	Zn(AzDC)(4,4'-BPE)$_{0.5}$ (20)	[BMIM][NTf_2]	ambient	ambient	3.99*
**ZIF-8-PL (30 wt%)[10]**	ZIF-8 (30)	[DBUPEG] [NTf_2]	1*	ambient	3.36*
**PoIL1-Z10[11]**	ZIF-8 (10)	[$P_{6,6,6,14}$][NTf_2]	0.99	30.14	1.90
**PoIL2-Z5[11]**	ZIF-8 (5)	[$P_{6,6,6,14}$][Cl]	1	30.13	1.90
**PoIL1-H5[11]**	HKUST-1	[$P_{6,6,6,14}$][NTf_2]	1	30.14	1.96
**Im-UiO-PL[12,+]**	Deim-UiO-66	PEGS	1*	25	11.87*
**UiO-66-liquid/[M2070][IPA][13]**	UiO-66 (50)	[M2070][IPA]	1	25	9.7
**PL7_10**	ZIF-7 (10)	Silicone 704	1.2	25	5.24
**PL7_5**	ZIF-7 (5)	Silicone 704	1.2	25	3.12
**PL9_10**	ZIF-9 (10)	Silicone 704	1.2	25	3.53
**PL8_5**	ZIF-8 (5)	Silicone 704	1.2	25	1.94
**PL-ii-2-Mg_2(dobpdc)**	Mg_2(dobpdc) (5)	Silicone 704	1.2	25	4.73

* these values are based on optical observation of the relevant graphs provided in the literature.

+ Notice, this material has a melting point around room temperature, so that the viscosity of the liquid is expected to be very high and not comparable to the other PLs documented here.

Nomenclature of the solvents acronyms used in Supplementary Table 2: Genosorb 1753 = $CH_3(OCH_2CH_2)_nOCH_3$, n = 4–10, [$P_{6,6,6,14}$][NTf_2] = trihexyltetradecylphosphonium bis(trifluoromethylsulfonyl)imide, [$P_{4,4,4,4}$][OAc] = tetrabutylphosphonium acetate, [$P_{4,4,4,4}$][Lev] = tetrabutylphosphonium levulinate, [BPy][NTf_2] = pyridinium bis(trifluoromethanesulfonyl)imide, [BMIM][NTf_2] = 1-butyl-3-methylimidazolium bis(trifluoromethylsulfonyl)imide, [$P_{6,6,6,14}$][Cl] = trihexyltetradecylphosphonium chloride, PEGS : poly(ethylene glycol) sulfonate, [M2070][IPA] = polyethylene-polypropylene glycol monomethylether(2-propyl) ammonium isophthalate

Supplementary References

1. Park KS*, et al.* Exceptional chemical and thermal stability of zeolitic imidazolate frameworks. *Proc Natl Acad Sci USA* **103**, 10186-10191 (2006).

2. Klein RA*, et al.* Structural resolution and mechanistic insight into hydrogen adsorption in flexible ZIF-7. *Chem Sci* **12**, 15620-15631 (2021).

3. McDonald TM, Lee WR, Mason JA, Wiers BM, Hong CS, Long JR. Capture of Carbon Dioxide from Air and Flue Gas in the Alkylamine-Appended Metal–Organic Framework mmen-Mg2(dobpdc). *J Am Chem Soc* **134**, 7056-7065 (2012).

4. Horike S, Shimomura S, Kitagawa S. Soft porous crystals. *Nature Chemistry* **1**, 695-704 (2009).

5. Lemmon EW, McLinden MO, Friend DG. Thermophysical properties of fluid systems. *NIST Chemistry WebBook, NIST Standard Reference Database Number 69*, (2010).

6. Tsang MY*, et al.* Porous liquids as solvents for the economical separation of carbon dioxide from methane. *Materials Today* **60**, 9-16 (2022).

7. Avila J*, et al.* High-Performance Porous Ionic Liquids for Low-Pressure CO_2 Capture. *Angew Chem Int Ed* **60**, 12876-12882 (2021).

8. Kai A*, et al.* Modular Type III Porous Liquids Based on Porous Organic Cage Microparticles. *Adv Funct Mater* **31**, 2106116 (2021).

9. Brand MC, Rankin N, Cooper AI, Greenaway RL. Shining a Light on Photoresponsive Type III Porous Liquids. *Chem Eur J* **29,** e202202848 (2023)

10. Shan W*, et al.* New Class of Type III Porous Liquids: A Promising Platform for Rational Adjustment of Gas Sorption Behavior. *ACS Appl Mater Interfaces* **10**, 32-36 (2018).

11. Avila J, Cervinka C, Dugas PY, Padua AAH, Gomes MC. Porous Ionic Liquids: Structure, Stability, and Gas Absorption Mechanisms. *Adv Mater Interfaces* **8**, 2001982 (2021).

12. Zou Y-H*, et al.* Porous Metal–Organic Framework Liquids for Enhanced CO_2 Adsorption and Catalytic Conversion. *Angew Chem Int Ed* **60**, 20915-20920 (2021).

13. Zhao X*, et al.* A polyether amine modified metal organic framework enhanced the CO2 adsorption capacity of room temperature porous liquids. *Chem Commun* **55**, 13179-13182 (2019).
